# The clinical characteristics and outcomes of *Campylobacter* spp. bloodstream infection: A systematic review and meta-analysis

**DOI:** 10.1007/s10096-026-05465-w

**Published:** 2026-03-13

**Authors:** María Paniagua-García, José Manuel Bernal, María Pilar Sánchez-Suero, Jerónimo Pachón, María Eugenia Pachón-Ibáñez, Antonio J. Vallejo-Vaz, Elisa Cordero

**Affiliations:** 1 Clinical Unit of Infectious Diseases, Microbiology and Parasitology, Instituto de Biomedicina de Sevilla (IBiS), Virgen del Rocío University Hospital, CSIC/University of Seville, Seville, Spain; 2 CIBER de Enfermedades Infecciosas, Instituto de Salud Carlos III (CIBERINFEC, ISCIII), Madrid, Spain; 3 Institute of Biomedicine of Seville, Virgen del Rocío University Hospital, CSIC/University of Seville, Seville, Spain; 4 Department of Medicine, Faculty of Medicine, University of Seville, Seville, Spain; 5 Clinical Epidemiology and Vascular Risk, Instituto de Biomedicina de Sevilla, IBiS/Hospital Universitario Virgen del Rocío, Universidad de Sevilla/CSIC, Seville, Spain; 6 CIBER de Epidemiología y Salud Pública, Instituto de Salud Carlos III (CIBERESP, ISCIII), Madrid, Spain

**Keywords:** Bloodstream infection, *Campylobacter* spp., Immunosuppression, Primary immunodeficiency, Humoral immunodeficiency, Relapses

## Abstract

**Purpose:**

*Campylobacter* spp. bloodstream infection (BSI) poses a significant challenge in clinical practice, particularly among certain populations such as immunocompromised individuals, mainly humoral immunodeficiencies. Objectives are to provide a comprehensive assessment and summarise the current evidence of the characteristics, clinical management and outcomes associated with *Campylobacter* spp. BSI.

**Methods:**

We searched PubMed, EMBASE and Cochrane for original research articles and case series/reports published in English or Spanish between January-1990 and October-2024 including patients with *Campylobacter* spp. BSI. Outcomes included antimicrobial treatment received, treatment resistance, all-cause mortality and relapses. Case reports/series and primary research articles were summarised separately. Primary research studies were pooled in a random-effect meta-analysis to estimate mortality and relapse, overall and by subgroup species. Heterogeneity was assessed using the I^2^ statistic. Meta-regression for the relationship of outcomes and variables of interest was performed where data allowed. The quality of the studies was assessed using the NIH Quality Assessment Tool for Case Series Studies.

**Findings:**

From the 1441 articles retrieved from our search, 32 primary research articles (2138 participants) and 196 small case series or single case reports (212 cases) were included. Within the 32 retrospective studies, 1259 (58.9%) patients had some immunocompromise. The most frequent species was *Campylobacter jejuni* (*n* = 928, 43.4%), of which 47.8% were resistant to quinolones. The meta-analysis showed a mortality rate of 7.2% (95%CI 4.6%–10.2%; I^2^ 70.7%); subgroup meta-analysis: *C. jejuni*: 3.8%, *C. fetus*: 10.4%, *C. coli*: 10.1%. Meta-regression suggested a direct association between the proportion of *C. fetus* BSI episodes included in the studies and mortality. The pooled relapse rate was 2.3%, and meta-regression analysis indicated that *C. jejuni* infection was associated with a lower risk of relapse compared with *C. coli* and *C. fetus* (*p* = 0.0002).

**Interpretation:**

Our systematic review and meta-analysis suggest high antimicrobial resistance rates, mortality and risk of relapse, with some differences by *Campylobacter* species.

**Supplementary Information:**

The online version contains supplementary material available at 10.1007/s10096-026-05465-w.

## Introduction

*Campylobacter* spp. are Gram-negative bacilli transmitted to humans through contaminated food. They are the most common cause of bacterial enteritis in developed countries [1, 2]. However, *Campylobacter* spp. bloodstream infection (BSI) is a rare infection, complicating 1:100–1:1000 enteric episodes [3]. Recent studies have reported a 10-fold increase in the incidence of *Campylobacter* spp. BSI without increased detection in stool cultures [4, 5].

*Campylobacter* spp. BSI has been reported in male patients, those with advanced age, and those with a history of alcoholism, gastrointestinal surgery, human immunodeficiency virus (HIV) infection, chronic liver disease, haematological malignancies and solid neoplasia, or other immunocompromise diseases [6, 7]. Approximately half of all BSI episodes occur in immunocompromised patients [5, 8, 9], with a particularly high frequency in those with primary immunodeficiencies (PIDs) [10–12].

The *Campylobacter* spp. that cause BSI appears to have changed over time. In older studies, such as a French series of 183 cases of *Campylobacter* spp. BSI included up to 2004, *Campylobacter fetus* was the most common species [13]; however, in more recent studies from the last decade, *Campylobacter jejuni* and *C. fetus* were the most common followed by *Campylobacter coli*. In patients with hypogammaglobulinemia, *C. coli* was the most common of these species [12]. Little is known about whether certain *Campylobacter* spp. may cause BSI more frequently in specific hosts.

*Campylobacter* spp. BSI has been reported to be associated with an overall favourable outcome. However, mortality rates reported in different studies range from 2% to 28% [5, 14–16], compared with only 1–2% in patients with non-BSI *Campylobacter* spp. enteritis [3, 5, 17]. This high mortality rate may reflect the general frailty of patients with BSI, with a high prevalence of comorbidities and immunosuppressive treatment. In addition, relapse may be a significant problem for certain populations. Case reports and some retrospective studies suggest that relapse is particularly common in patients with PIDs [18, 19]. However, as few studies have systematically evaluated it, the role of different *Campylobacter* spp. in the risk of relapse is poorly understood.

One difficulty in treating this infection is antimicrobial resistance. Resistance to quinolones has increased over time, with rates exceeding 90% in some studies [3, 8, 12, 20]. The susceptibility of *Campylobacter* spp. isolates from blood cultures to macrolides has been high in previous studies [3, 8]; however, macrolide resistance may be increasing, as a recent study shows that almost a quarter of *C. coli* isolates are resistant to them [12]. Furthermore, unlike *Campylobacter*-associated enteritis, there is no consensus on the most appropriate treatment for *Campylobacter* spp. BSI. The wide heterogeneity of antibiotic regimens makes it very difficult to determine whether specific therapeutic approaches, such as combined antimicrobial therapy or longer courses of antibiotics, may improve survival or reduce recurrence.

To the best of our knowledge, no systematic reviews or meta-analysis have comprehensively assessed *Campylobacter* spp. BSI. *Campylobacter* BSI, especially by *C. coli*, appears to be an emerging disease, particularly in patients with humoral immunocompromise. Its high recurrence rate and increasing antimicrobial resistance make identifying and treating it challenging. To date, the available evidence is scarce and based on case reports and heterogeneous retrospective studies. The aim of this systematic review and meta-analysis was to assess the available evidence on *Campylobacter* spp. BSI, including risk factors, clinical findings, recurrence and mortality, as well as the potential differences in outcomes between patient populations.

## Methods

We conducted a systematic review and meta-analysis in accordance with the Preferred Reporting Items for Systematic Reviews and Meta-Analyses (PRISMA) guidelines [21], the checklist for which is shown in Supplemental Table S1. The review protocol was registered in the International Prospective Register of Systematic Reviews (PROSPERO) Database (registration number: CRD42024592832).

### Search strategy and selection criteria

We used a PECOS (population, exposure, comparison, outcomes, study design) model as a framework for defining our search strategy. Eligible studies were those that: (i) included patients with *Campylobacter* spp. BSI (regardless of the underlying type/infection/site of primary infection) and (ii) reported on at least one of the following outcomes: mortality, relapse, hospital admission, response to treatment and/or resistance to antimicrobial therapy. Research articles (any study design), case series and case reports were included, while conference abstracts were not. Reviews and other non-primary research articles were also excluded.

A search strategy was built using both MeSH terms (Emtree terms in the case of Embase) and free text (keywords, synonyms, and word variations) related to *Campylobacter*, species of *Campylobacter*, bloodstream infection and sepsis. The full search strategy is shown in Supplemental Table S2. We searched the electronic databases PubMed, Embase and Cochrane, from 01 January 1990 to 20 September 2024. The search was limited to articles in English and Spanish; but no other limits were applied.

### Study selection

**Fig. 1:**
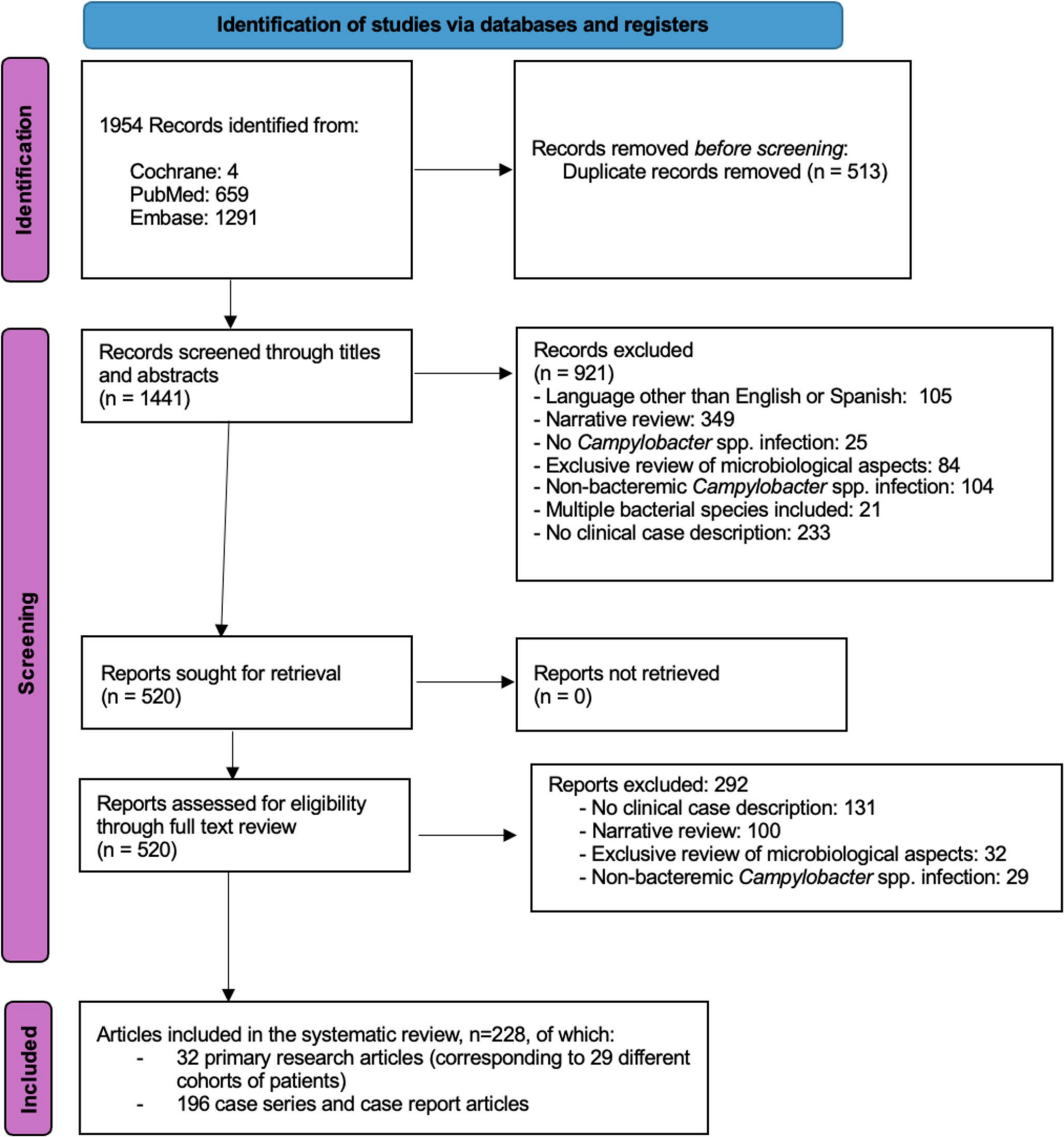
Preferred Reporting Items for Systematic Reviews and Meta-Analyses (PRISMA) flow diagram depicting the study selection for inclusion of articles in the systematic review

The literature search and article screening were performed independently by two investigators (J.M.B. and M.P.G.), with any disagreements resolved through discussion with a third reviewer (E.C.M.). The screening flow was conducted according to PRISMA recommendations [21], the details of which are shown in Fig. 1. Briefly, after discarding duplicates from the three databases, a total of 1441 articles were retrieved and subjected to screening through titles and abstracts. This step led to 520 articles that required full-text review to assess their eligibility. Finally, 228 articles met the inclusion criteria and were included in the systematic review. Of them, 32 articles, corresponding to 29 different cohorts of patients, were original research studies and 196 articles were cases series or reports.

### Data collection

Data were extracted independently by two reviewers (J.M.B. and M.P.G.), and both examined all extraction sheets to ensure accuracy. The data recorded included the design and characteristics of the studies, the characteristics of the participants/cohorts, and clinical and microbiological data related to *Campylobacter* spp. BSI, treatments, and outcomes, as shown in Tables 1, 2 and 3 and Supplemental Tables S4, S5, S6, S7, S8, S9, S10 and S11.

**Table 1:** Characteristics and design of the 32 included retrospective studies

Study	Country	Study period (years)	Study design	Source of data	Number of patients	Inclusion criteria	Exclusion criteria
Sunnerhagen et al., 2024 [17]	Sweden	2015–2022	Retrospective study	1 hospital	29	*Campylobacter* spp. BSI (compared to non-bacteraemia controls without BSI)	NR
Meda et al., 2024 [22]	India	2015–2021	Retrospective study	1 hospital	12	*Campylobacter* spp. BSI	NR
Baek et al., 2024 [23]	South Korea	2010–2021	Retrospective study	7 hospitals	108	*Campylobacter spp.* BSI	Age < 19 years old
Graham, 2024 [24]	England	2012–2021	Retrospective study	1 hospital	34	Laboratory confirmed *Campylobacter* spp. BSI	Missing National Health Service (NHS) numbers and personally identifiable information samples with missing specimen dates
Zayet et al., 2023 [25]	France	2000–2021	Retrospective study	1 hospital	21	*C. fetus* BSI infection in hospitalized patients	NR
Otsuka et al., 2023 [26]	Japan	2011–2021	Retrospective study	3 hospitals	39	*Campylobacter* spp. BSI	NR
Tinévez et al., 2023 [14]	France	2015–2019	Retrospective study	37 hospitals	57	*Campylobacter* spp. BSI and endovascular infection	NR
Tinévez et al., 2022 [12]	France	2015–2019	Retrospective study	37 hospitals	592	*Campylobacter* spp. BSI	NR
Tau et al., 2022 [5]	Israel	2007–2020	Retrospective case-control study.	1 hospital	76	*Campylobacter* spp. BSI > 18 years of age; non-bacteraemia *Campylobacter* infection as controls	Patients whose blood culture was not recovered
Shahunja et al., 2020 [27]	Bangladesh	2014–2017	Retrospective study	1 hospital	9	Children under 5 years of age admitted with diarrhea and *Campylobacter* BSI spp.	Positive blood cultures for: coagulase-negative *Staphylococcus*, *Corynebacterium* spp., *Micrococcus* spp. and *Candida* spp.
Liu et al., 2019 [28]	Taiwan	1998 − 2014	Retrospective study	1 hospital	56	*Campylobacter* spp. BSI	NR
O’Hara et al., 2017 [15]	United Kingdom	1970–2013	Retrospective study	1 hospital	41	*Campylobacter* spp. BSI	NR
Yamamoto et al., 2017 [29]	Japan	2008–2014	Retrospective case-control study.	1 hospital	14	*Campylobacter* or *Helicobacter* spp. BSI	NR
Marchand- Senécal et al., 2017 [30]	Canada	2014–2016	Retrospective study	1 hospital	3	*C. fetus* BSI	NR
Cypierre et al., 2014 [31]	France	2007–2013	Retrospective study	1 hospital	20	*C. fetus* BSI	NR
Mori et al., 2014 [32]	Japan	2001–2013	Retrospective study	1 hospital	7	*C. jejuni* BSI	NR
Ben-Shimol et al., 2013 [10]	Israel	1989–2010	Retrospective study	1 hospital	60	*Campylobacter* spp. BSI < 19 years of age	Isolation of *Campylobacter* spp. from the same patient less than 30 days apart
Liao et al., 2012 [9]	Taiwan	1998–2008	Retrospective study	1 hospital	24	*Campylobacter* spp. BSI	NR
Feodoroff et al., 2011 [16]	Finland	1998–2007	Retrospective study	National Infectious Diseases Register	76	*Campylobacter* spp. BSI	NR
Fica et al., 2011 [33]	Chile	1986–2010	Retrospective study	1 hospital	6	*Campylobacter spp*. BSI. > 15 years of age	NR
Nielsen et al., 2010 [3]	Denmark	1995–2004	Retrospective study	NR	46	*Campylobacter* spp. BSI	NR
Fernández-Cruz et al., 2010 [34]	Spain	1985–2007	Retrospective study	NR	68	*Campylobacter* spp. BSI	Isolation of *Campylobacter* spp. from the same patient less than 30 days apart
Cochennec et al.,2008 [35]	France	1997–2006	Retrospective study	NR	5	*Campylobacter fetus* BSI and infected arterial aneurysm	NR
Gazaigne et al., 2008 [7]	France	1998–2006	Retrospective study	1 hospital	21	*Campylobacter fetus*. BSI	NR
Pacanowski et al., 2008 [13]	France	2000–2004	Retrospective study	23 hospitals	178	*Campylobacter* spp. BSI	NR
Woo et al.,2002 [36]	China	1996–2000	Retrospective study	NR	8	*Campylobacter fetus* BSI	NR
Tee et al., 1998 [37]	Australia	1985–1995	Retrospective study	1 hospital	21	*Campylobacter* spp. BSI	NR
Pigrau et al., 1997 [38]	Spain	1979–1996	Retrospective study	1 hospital	58	*Campylobacter* spp. BSI	NR
Font et al., 1997 [39]	Spain	1987–1994	Retrospective study	1 hospital	30	*Campylobacter* spp. BSI > 14 years age	NR
Pigrau et al., 1996 [40]	Spain	1979–1994	Retrospective study	1 hospital	7	*Campylobacter* spp. BSI in HIV infected patients	NR
Schonheyder et al., 1995 [41]	Denmark	1989–1994	Retrospective study	1 hospital	15	*Campylobacter* spp. BSI	NR
Skirrow et al., 1993 [42]	United Kingdom	1981–1991	Retrospective study	NR	394	*Campylobacter* spp. BSI	NR

*NR* not reported

**Table 2:** Distribution of extra-intestinal manifestations of *Campylobacter* BSI, extracted from the 32 included retrospective studies

Extraintestinal manifestations of Campylobacter spp. BSI	TOTAL* N = 1311
Endovascular infection (global), n (%) - Endovascular, n (%) - Mycotic aneurism, n (%) - Endocarditis, n (%)	84 (6.4) - 53 (4) - 18 (1.4) - 13 (0.9)
Soft tissue, n (%)	64 (4.9)
Bone/joint infection, n (%) - Septic arthritis, n (%)	42 (3.2) - 3 (0.2)
Pneumonia, n (%)	31 (2.4)
Peritonitis, n (%)	10 (0.8)
Meningitis, n (%)	9 (0.7)
Infected medical device, n (%)	8 (0.6)
Cholangitis, n (%)	2 (0.2)
Thrombophlebitis, n (%)	2 (0.2)
Pancreatitis, n (%)	1 (0.1)

*For those where information is available

**Table 3:** Distribution of antibiotic families used in monotherapy and combination therapy

Antibiotic groups used in monotherapy	TOTAL *n* = 61*
Quinolones, n (%)	20 (32.8)
Cephalosporin, n ((%)	18 (29.5)
Amoxicillin–clavulanic acid, n (%)	13 (21.3)
Macrolides, n (%)	3 (4.9)
Amoxicillin, n (%)	3 (4.9)
Carbapenem, n (%)	3 (4.9)
Pristinamycin, n (%)	1 (1.6)
Tigecycline, n (%)	0 (0)
**Antibiotic groups used in combination therapy.**	**TOTAL** n = 96*
ß-lactam + aminoglycoside, n (%)	42 (43.8)
ß-lactam + quinolone, n (%)	29 (30.2)
ß-lactam + macrolide, n (%)	9 (9.4)
Metronidazole + ß-lactam, n (%)	5 (5.2)
Macrolide + quinolone + aminoglycoside, n (%)	3 (3.1)
ß-lactam + vancomycin, n (%)	2 (2.1)
Quinolone + aminoglycoside, n (%)	2 (2.1)
Tetracycline + macrolide + aminoglycoside, n (%)	1 (1.0)
Tetracycline + macrolide, n (%)	1 (1.0)
Metronidazole + quinolone, n (%)	1 (1.0)
Quinolone + ß-lactam + aminoglycoside, n (%)	1 (1.0)
Macrolide + quinolone, n (%)	0 (0)
Macrolide + ß-lactam+ aminoglycoside, n (%)	0 (0)

*For those where information is available.

### Quality assessment of the included studies

The quality (risk of bias) of the primary research articles was assessed independently by two investigators (J.M.B. and M.P.G.). They used the NIH Quality Assessment Tool for Case Series Studies [43], taking into account items related to research questions/objectives, population descriptions, interventions, results and outcomes, as detailed in Supplemental Table S3. The tool was not applied to case reports; by definition, all case reports were considered to have a high risk of bias and to provide less evidence than primary research articles. The NIH tool was selected due to its relative ease of application and our prior experience with it, whereas alternative tools, such as ROBINS or the Joanna Briggs Institute tool, are either more complex or less familiar.

### Data analysis

All studies were included in the systematic review, but data were presented and described separately for primary research studies and for case series and reports. In the case of meta-analyses, only primary research studies were entered in the analysis; additionally, if the same cohort of patients (in full or in part) was included in different articles, only one of them was included in each set of meta-analysis to avoid entering the same patients twice in the same analysis; in these cases, article selection was carried on a case-by-case basis, considering the number and type of participants, data provided or quality.

The results of the systematic review are shown descriptively tabulated, as reported by the original studies. Meta-analyses were performed to obtain a pooled estimate across the studies for the outcomes of interest, namely the proportion of mortality associated with *Campylobacter* spp. BSI and relapse. Individual study effect size confidence intervals of 95% (95% CIs) were obtained from the sample size and proportion of cases with the outcome in each study using the Clopper–Pearson method. The Freeman–Tukey double arcsine transformation was applied to the primary study data before meta-analysis. The final results (estimate and 95% CI) were backtransformed. The meta-analyses were performed using a random-effects model (inverse variance method), and the I^2^ statistic (95% CI) was used to assess between-study heterogeneity.

Small study effects/publication biases were graphically assessed using funnel plots, and asymmetry was further formally examined using Peter’s regression test. Where there were suggestions of asymmetry, we applied the Duval and Tweedie trim-and-fill method to adjust for funnel plot asymmetry (bias-corrected estimate of the pooled effect size). An assessment of potentially influential studies was performed by searching for outlier studies using the function “find.outliers” (dmetar R package)” and excluding these when re-conducting the meta-analysis.

We conducted subgroup meta-analysis based on the species of *Campylobacter*. Meta-regression analyses were performed where the reported data allowed, particularly based on the proportion of patients in each study with BSI due to different *Campylobacter* spp. Age- and sex-adjusted meta-regression was applied in these cases. Since age was reported using means in some studies and medians in others, we assumed the variable age by including all studies combining both types of measure; results from conducting meta-regression separately for studies reporting age as means and for studies reporting age as medians were consistent and in the same direction.

Meta-analyses were conducted with R version 4.2.3 (through RStudio v2023.03.0) [44] using the “meta” package [45].

### Ethics

This study relies on data already published in the aforementioned public electronic databases and uses aggregated data; therefore, approval by a research ethics committee was unnecessary.

## Results

### Systematic review of primary research studies

A total of 32 original research articles (all retrospective studies) from 1990 to 2024 were examined in the systematic review, which included 2138 patients with *Campylobacter* spp. BSI [3, 5, 8–11, 13–17, 22–24, 26–30, 32–34, 36–45],. Full characteristics of these studies are shown in Tables 1 and 2 and Supplementary Tables S4, S5 and S6. Most studies were from France (*n* = 7) [8, 11, 13, 14, 43–45], Spain (*n* = 4) [34, 38–40], Japan (*n* = 3) [26, 29, 32] and the UK (*n* = 3) (Table 1).

In the 29 studies reporting sex, 1154 (71.6%) patients were males. The median age was 55.9 years (IQR 46–68.7). A total of 1259 (58.9%) cases occurred in somewhat immunocompromised patients. Of these, 221 (10.3%) patients received corticosteroids and 280 (13.1%) were treated with other immunosuppressive drugs, including chemotherapy. Additionally, 336 (15.7%) patients had active solid neoplasia, 259 (12.1%) had hematological malignancies, and fewer than 5% of the patients had other immunocompromise conditions (Supplementary Table S4).

According to the available clinical information, fever was reported in 1187 (75.6%) out of 1570 and diarrhea in 885 (42.9%) out of 1990 cases. Secondary sources of infection or extra-intestinal complications were reported in 19 of the 32 studies (*n* = 1311 cases). In total, there were 256 (19.5%) episodes of extra-intestinal infection, mainly endovascular infection (*n* = 84, 6.4%), including mycotic aneurysms, and endocarditis, followed by soft tissue infection (*n* = 64, 4.9%), bone and joint infection (*n* = 42, 3.2%), pneumonia (*n* = 31, 2.4%), peritonitis (*n* = 10, 0.8%), meningitis (*n* = 9, 0.7%), infected medical device (*n* = 8, 0.6%) and cholangitis (*n* = 2, 0.2%) (Table 2).

The most isolated species was *C. jejuni* (*n* = 928, 43.4%), followed by *C. fetus* (*n* = 581, 27.2%) and *C. coli* (*n* = 200, 9.3%). *Campylobacter* spp. were isolated from stools in 24.8% (*n* = 231) of the 931 episodes in which samples were collected.

The resistance analysis performed in the different studies was very heterogeneous and not all episodes included susceptibility testing. Among the *C. jejuni* isolates, 47.8% were resistant to quinolones (*n* = 241/504), 3.5% to macrolides (*n* = 14/406) and 1.5% to amoxicillin-clavulanic acid (*n* = 6/400). For *C. fetus*, the resistance rates were 24.6% to quinolones (110/447) and 3.5% to macrolides (15/435), while no resistance was observed for amoxicillin-clavulanic acid (0/9) or carbapenems (0/9) (Supplemental Table S5).

In the 633 episodes with available information, 409 (64.6%) received a single antibiotic and 224 (35.4%) received two or more (Supplemental Table S6). The distributions of the antibiotic families used in both mono- and combination therapies are detailed in Table 3. Information about specific antimicrobials was only available for 61 patients treated with monotherapy and 96 patients receiving combination antimicrobial therapy. Quinolones were the most used in monotherapy (*n* = 20, 32.8%), followed by cephalosporins (*n* = 18, 29.5%), and amoxicillin-clavulanic acid (*n* = 13, 21.3%). Macrolides were rarely used as monotherapy; this was seen in only three cases. The most common combination therapy was a ß-lactam plus an aminoglycoside (*n* = 42, 43.8%). The median length of hospitalization was 17.8 days (IQR 8.1–27.6).

### Quality of studies assessment (primary research studies)

Results of the assessment of the 32 articles are presented in Supplemental Table S3. Overall, there was low risk of bias in most studies in most items evaluated, with the exception of the couple of questions related to the way of inclusion of participants and controls where applicable, which revealed high risk of bias in 14 studies (non-consecutive inclusion of participants and/or subjects not fully comparable where applicable).

### Meta-analysis of Campylobacter spp. BSI outcome

**Fig. 2:**
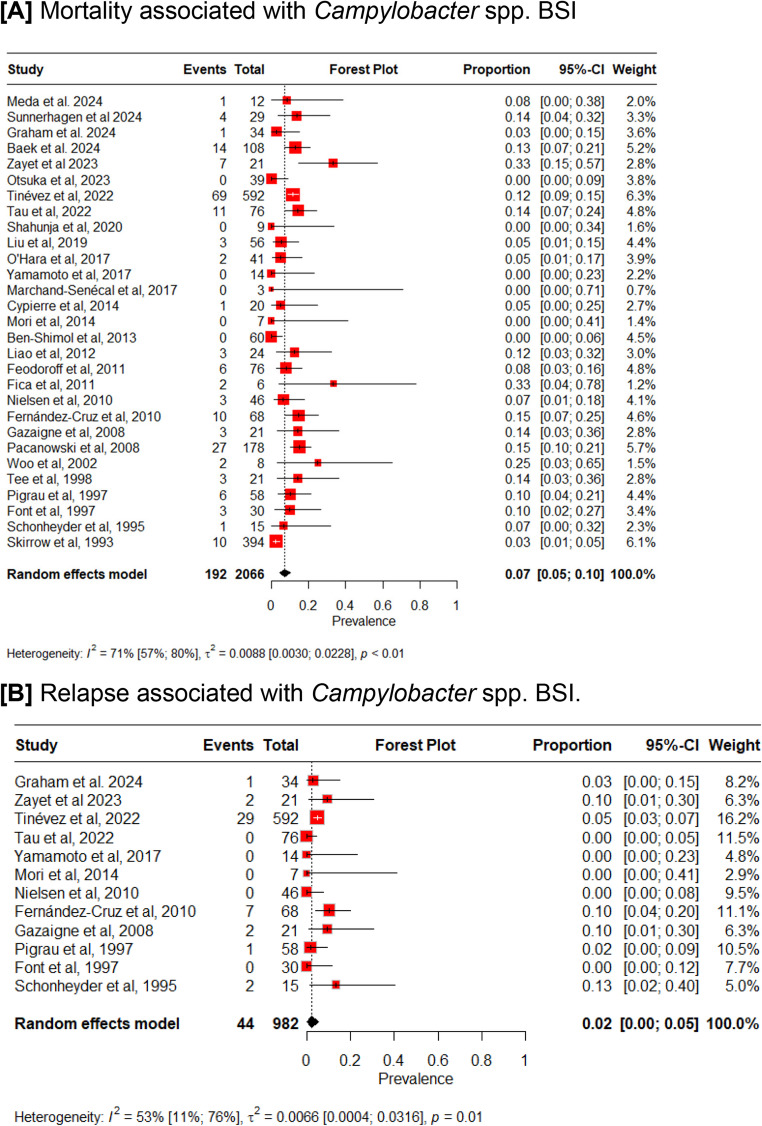
Mortality (panel **A**) and relapse (panel **B**) associated with *Campylobacter*ssp*.* bloodstream infections (BSI). Proportion estimates are shown as number of cases in 1 (toobtain percent, multiply by 100)

A total of 29 different studies, comprising 2066 participants with *Campylobacter* spp. BSI (192 death events), were included in the meta-analysis assessing mortality. The pooled estimate proportion of mortality was 7.2% (95%CI 4.6%, 10.2%; I^2^ 70.7%) (Fig. 2A). A funnel plot was built to assess potential small-study effects/publication bias (Supplemental Figure S1). Graphically speaking, there was no obvious asymmetry; however, a formal statistical test suggested plot asymmetry (Peter’s regression test *p* = 0.0199). To adjust for funnel plot asymmetry and calculate a bias-corrected pooled estimate, we applied the trim-and-fill method. This procedure filled the meta-analysis with two additional studies and resulted in a pooled estimate mortality of 6.3% (95%CI 3.7%, 9.4%, I^2^ 72.2%) (Supplemental Figure S2), suggesting a slight overestimation of the proportion of mortality in the original meta-analysis (7.2% vs. 6.3%). On the other hand, the assessment of potentially influential studies found four outliers [10, 25, 26, 42]; the exclusion of these resulted in a higher pooled estimate of mortality than the original meta-analysis: 9.5% (95%CI 7.9%, 11.2%), while leading to no significant heterogeneity (I^2^ 0.0%, 95%CI 0.0%, 43.9%, *p* = 0.5295).

**Fig. 3:**
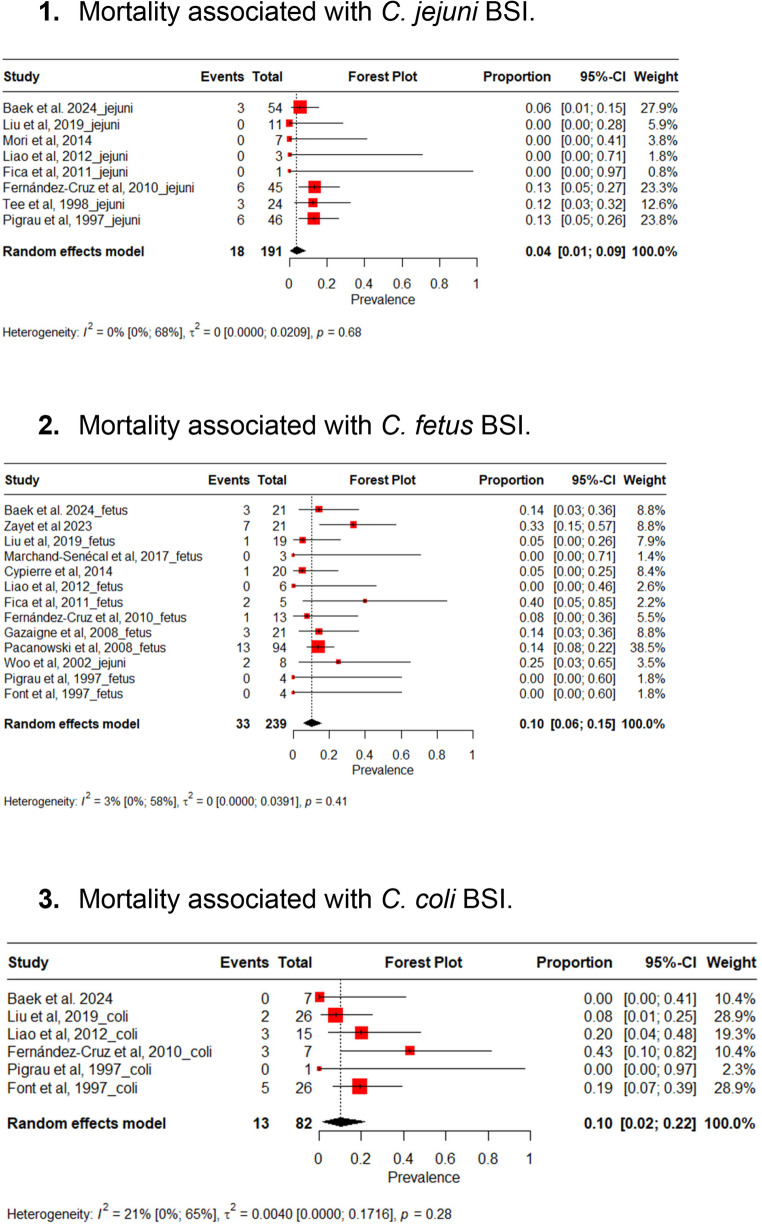
Subgroup meta-analysis for the mortality associated with *Campylobacter* bloodstream infection based on the *Campylobacter* spp. Proportion estimates are shown as number of cases in 1 (to obtain percent, multiply by 100). p-value for subgroup comparison, p=0.2573

**Fig. 4:**
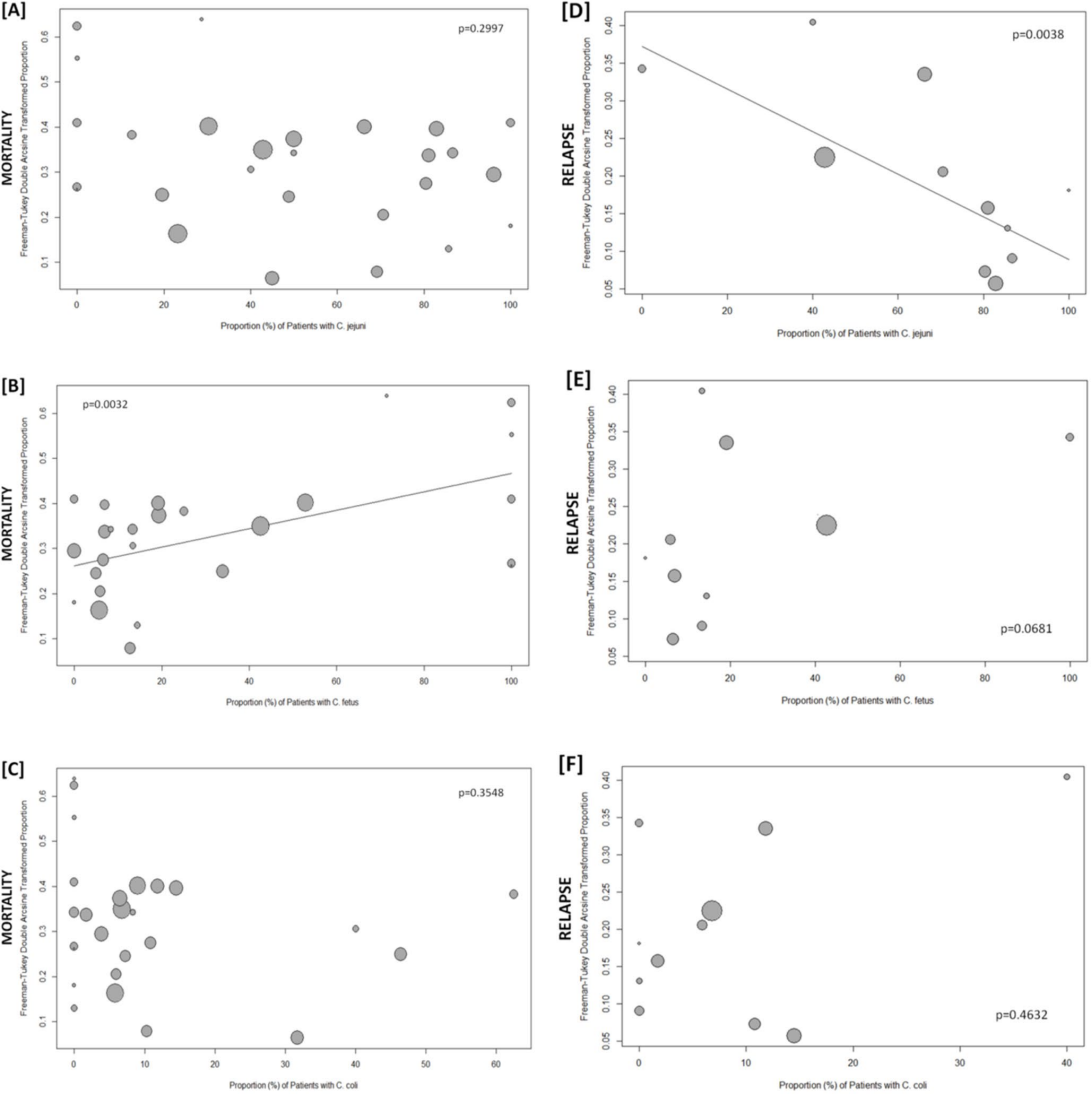
Meta-regression analysis for the mortality (panels **A**-**C**) and relapse (panels **D**-**F**) associated with Campylobacter bloodstream infection based on the proportion of participants included in the studies with C. jejuni (panels **A **& **D**), C. fetus (panels **B **& **E**) and C. coli (panels**C **& **F**) episodes

Subgroup meta-analysis by *Campylobacter* spp. included a total of 8, 13 and 6 studies that reported mortality specifically related to *C. jejuni*, *C. fetus* and *C. coli* BSI, respectively. The pooled proportion of mortality associated with *C. jejuni* was 3.8% (95%CI 0.6%, 8.6%; I^2^ 0.0%), with *C. fetus* at 10.4% (95%CI 6.0%, 15.5%; I^2^ 3.3%), and with *C. coli* at 10.1% (95%CI 1.9%, 21.7%; I^2^ 20.7%); p-value for subgroup comparison, *p* = 0.2573 (Fig. 3). Heterogeneity was not significant in all cases. Meta-regression analyses (24 studies) revealed a direct relationship between the proportion of participants with *C. fetus* BSI included in the studies and the mortality observed in the studies (age- and sex-adjusted *p* = 0.0019, estimate 0.002 [95%CI 0.001, 0.004], residual heterogeneity I^2^ 0.13%); no significant relationship was found in cases of *C. jejuni* or *C. coli* BSI (age- and sex-adjusted *p* = 0.0701 and *p* = 0.8898, respectively) (Fig. 4A and C, Supplemental Table S7).

Twelve studies reported the occurrence of relapse (982 participants, 44 relapse events). The pooled estimate for the proportion of relapse was 2.3% (95%CI 0.38%, 5.4%; I^2^ 53.5%) (Fig. 2B). A funnel plot is shown in Supplemental Figure S3; regression test suggested plot asymmetry (*p* = 0.0359); however, when we applied the trim-and-fill method, no additional studies were added. An assessment of influential studies found one potential outlier [5]; after excluding this study, the meta-analysis resulted in a proportion of relapse of 3.1% (95%CI 0.93%, 6.1%; heterogeneity I^2^ 37.1% [95%CI 0.0%, 69.1%], *p* = 0.1028). Subgroup analysis for the occurrence of relapse based on the *Campylobacter* spp. was not possible due to the lack of sufficient reported data in the studies. Finally, meta-regression analyses found an indirect relationship between the proportion of participants with *C. jejuni* BSI included in the studies and the occurrence of relapse observed in the studies (the higher the proportion of participants with *C. jejuni* BSI, the lower the occurrence of relapse; age- and sex-adjusted p-value 0.0002, estimate − 0.004 [95%CI − 0.006, − 0.002], residual heterogeneity I^2^ 0.0%). No significant relationship was found in cases of *C. fetus* or *C. coli* BSI (age- and sex-adjusted, *p* = 0.1179 and *p* = 0.3472) (Fig. 4D and F, Supplemental Table S7).

### Systematic review of case series and case reports

A total of 196 case series or single case reports were assessed (for references, see Supplemental Material), including 212 cases with *Campylobacter* spp. BSI. Full details are reported in supplementary material (Tables S8, S9, S10 and S11 and supplementary text 1).

Most patients were male (*n* = 134, 63.2%), with a median age of 54 years (interquartile range [IQR] 33–70). Patients with BSI caused by *C. fetus*, compared to those with BSI caused by *C. coli* and *C. jejuni*, were older (median ages of 63.5 years [IQR 46.25–75], 40.5 [IQR 32–57.75.75] and 36.5 [IQR 32–57.75.75], respectively), and more frequently suffered with diabetes mellitus (20.2%, 0% and 6.8%, respectively). Patients with *C. fetus* and *C. jejuni* BSI had more comorbidities compared to those with *C. coli* BSI (70.8%, 75.7% and 54.5%, respectively).

Patients with *C. coli* and *C. jejuni* BSI more frequently had primary or secondary immunocompromise compared to those with *C. fetus* BSI (59.1%, 64.9% and 32.6%, respectively). Primary or secondary humoral deficiencies were also more frequent in patients with *C. coli* and *C. jejuni* BSI compared to those with *C. fetus* BSI (40.9%, 41.9%, and 18%, respectively). *C. coli* predominated in patients with agammaglobulinemia compared to *C. jejuni* (22.7% and 12.2%, respectively). No cases of *C. fetus* BSI were observed in patients with agammaglobulinemia (Supplemental Table S8).

Fever was present in 158 (74.5%) patients, with no differences regarding *Campylobacter* spp. Diarrhea was more frequent in patients with *C. coli* and *C. jejuni* compared to those with *C. fetus* (31.8%, 39.2% and 22.5%, respectively). Extra-intestinal manifestations, and specifically endovascular manifestations (endocarditis or other vascular infections), as well as neurological manifestations, meningitis and osteomyelitis, were more commonly reported among patients with *C. fetus* BSI. In 40 (54.8%) patients, *Campylobacter* spp. were also isolated from feces (from 73 patients where this information was available), at rates of 70% in *C. coli*, 64.5% in *C. jejuni* and 40% in *C. fetus* episodes.

Antimicrobial resistance rates were heterogeneously reported. Amoxicillin resistance was higher in *C. jejuni* and *C. coli* than *C. fetus* (20%, 47.7% and 0%, respectively). Quinolone resistance was common, with a higher proportion in *C. jejuni* isolates, compared to *C. coli* and *C. fetus* (70.9%, 54.6 and 33.3%, respectively) (Supplemental Table S9).

A total of 102 episodes (48.1%) received only one antibiotic and 88 (41.5%) were treated with combined antimicrobial therapy. Patients with *C. coli* and *C. fetus* BSI received longer courses of combined treatment compared to those with *C. jejuni* cases (35 [IQR 21–57] vs. 35 [IQR 19.5–42] vs. 14 [IQR 7–21] days, respectively).

Global 30-day mortality was 8.8%, and higher in the case of *C. jejuni* (11.1%) compared to *C. fetus* (7.1%) and *C. coli* (0%). Relapses were more frequent in BSI episodes of *C. coli* (35%) than of *C. jejuni* (17.2%) or *C. fetus* (4.9%) (Supplemental Table S8).

In summary, analysis of individual cases revealed two distinct clinical–microbiological patterns in *Campylobacter* BSI, corresponding to the species involved. *C. fetus* BSI affected older patients, was less frequently associated with immunosuppression, more often involved extraintestinal disease, showed universal susceptibility to amoxicillin, and had lower relapse rates. In contrast, *C. coli* and *C. jejuni* BSIs were more common in immunocompromised patients, were more frequently associated with diarrhea and fecal isolation, showed higher resistance to amoxicillin and quinolones, and had significantly higher relapse rates.

When cases were considered according to their immunological status (Supplemental Table S10), patients with PIDs were younger than those without (median age 26 [IQR 18.5–44.7] vs. 57 [IQR 38–71] years, *p* < 0.001) and had fewer different comorbidities (32% vs. 77.4%, *p* < 0.001). Specifically, diabetes mellitus was significantly more frequent among non-PID patients (*n* = 23, 14.5%) compared to PID patients PID (*n* = 0), p value = 0.042. Regarding clinical characteristics, patients with PIDs were more likely to have respiratory symptoms such as cough (20% vs. 6.9%, respectively, *p* = 0.003) and skin symptoms (36% vs. 11.9%, respectively, *p* = 0.008). In general, 60% of patients with PIDs had extra-intestinal manifestations compared to 45.3% in non-PID patients. *Campylobacter* spp. were also isolated from feces in 10 (71.4%) patients with PID, compared to 44% of those without PIDs (*n* = 22).

While *C. coli* and *C. jejuni* were significantly more common in patients with PIDs (28% and 52% of the total of isolates), *C. fetus* was the most common species in non-PID patients (49.7%). In general, isolates from patients with PIDs had higher rates of antimicrobial resistance, this was especially relevant in the case of macrolides, with 61.5% of isolates (*n* = 8) resistant to macrolides, compared with 12.1% (*n* = 7) in non-PID patients (*p* < 0.001) (Supplemental Table S11).

There were no significant differences in treatment-related variables between patients with and without PID; however, the wide variability of antimicrobial schedules reported must be noted. In terms of outcomes, 30-day mortality was higher in non-PID patients: at 10.7% vs. 0%, while all-cause mortality was similar in both groups (8.3% vs. 12.7%, respectively). On the other hand, relapses were significantly more common among PID patients (65.2% vs. 5.6%, respectively, *p* < 0.001). Among the 13 patients in whom macrolide resistance was tested, nine experienced relapse: seven (77.8%) in the presence of macrolide resistance and two (22.2%) in the absence of macrolide resistance (*p* = 0.07).

## Discussion

Managing *Campylobacter* spp. infection can be challenging due to the lack of good-quality evidence, variable rates of antimicrobial resistance and the infection’s ability to relapse, particularly in immunocompromised patients. In this study, which represents the first systematic review and meta-analysis of *Campylobacter* spp. BSI, a total of 228 publications were selected, including 32 primary research studies (all retrospective cohort studies) and 196 case series or single case reports, comprising a total of 2138 and 212 *Campylobacter* spp. BSI episodes, respectively. The results show that these infections are associated with considerable mortality, especially those episodes caused by *C. fetus*, and that there is a high rate of relapses in patients with PIDs.

The first aspect to consider is that *Campylobacte*r spp. BSI mostly occurs in individuals with underlying conditions, despite the fact that the median age of patients is not high (55.9 years). Sunnerhagen et al. [17]. reported that patients with *Campylobacter* spp. BSI were older and had more comorbidities than patients with *Campylobacter* enteritis without BSI. Diabetes mellitus, liver disease, solid neoplasia, and haematologic malignancies were among the most frequent underlying conditions, as stated in other studies [26, 38, 42]. In this systematic review, immunocompromising conditions were the most common comorbidity found in patients with *Campylobacter* spp. BSI. Nearly 60% of episodes occurred in patients with some type of immunocompromise, such as solid neoplasia or haematologic malignancies, solid organ and haematopoietic stem cell transplant, immunosuppressive drugs, and PIDs, among others. Primary humoral immunodeficiencies, mainly agammaglobulinemia, were especially common in the small cases series and case report studies, which is noteworthy given that PIDs, and specially agammaglobulinemia, are considered rare diseases [46]. Most PIDs were humoral immunodeficiencies. However, the presence of PIDs or hypogammaglobulinemia was rarely reported in the retrospective cohorts included in this systematic review. These marked differences in the findings between retrospective series and single case reports may be related to a reporting bias in the single case publications or to the fact that PIDs were not specifically considered when designing the studies or reporting the results of some retrospective cohort studies.

Regarding clinical presentation, fever was the predominant symptom, occurring in three out of five patients, while gastrointestinal symptoms, commonly associated with *Campylobacter* spp. enteritis [12, 23, 26, 34], were less frequent. The presence of extra-intestinal disease, mainly endovascular and cellulitis, followed by bone/joint infection and pneumonia, in nearly 20% of cases, highlights this microorganism’s ability to disseminate in susceptible hosts [12, 25, 34].

In line with other studies [5, 22–24, 34], the most frequently isolated species was *C. jejuni*, followed by *C. fetus* and *C. coli*. However, species distribution varies according to the underlying condition of the patients: *C. coli* predominated in patients with humoral PIDs, especially in patients with agammaglobulinemia, while *C. fetus*, mostly according to small case series and case reports, occurred in older patients and patients with other comorbidities different from immunosuppression, and were more likely to have extra-intestinal infection, mainly endocarditis and endovascular infection, as suggested by previous studies [7, 13].

The *Campylobacter* app. antimicrobial resistance analysis performed was very heterogeneous across different studies. However, resistance, particularly to quinolones and macrolides, remains a significant concern. Quinolone resistance was higher in *C. jejuni* isolates causing BSI compared to *C. coli* and *C. fetus.* Among case reports with PIDs more than 60% of them were resistant to macrolides and fluoroquinolones; however, nearly all the isolates remained susceptible to carbapenems. These data from small case series and case reports should be considered cautiously, as they likely overestimate the incidence of resistance (reporting bias). It should be noted that susceptibility testing for carbapenems was rarely performed, mainly due to the lack of standardized susceptibility breakpoints, which may limit the interpretation of the results.

The overall mortality from *Campylobacter* spp. BSI from the meta-analysis was 6.3–7.2%. Mortality rates in individual studies were variable, ranging from 0 to 30% [12, 17, 39]. Meta-regression analyses showed a direct association between the proportion of participants with *C. fetus* BSI included in the studies and the associated mortality, as suggested in previous studies [7, 47, 48]. This could be explained by the different profiles of patients with *C. fetus* BSI, with more comorbidity and vascular complications.

Relapses are an important problem in *Campylobacter* spp. BSI occurring in patients with PIDs, particularly in cases of humoral immunodeficiencies, which are rare in patients with non-humoral PIDs [6, 19, 49]; this problem is possibly related to the immunocompromised hosts’ inability to eradicate the pathogen. In the single cases reported, we observed that recurrences occurred in two-thirds of PID patients while they occurred in only 5% of non-PID patients. In the actual meta-analysis, the proportion of relapses was 2.3%. The risk factors associated with relapse could not be analysed because this endpoint was not specified in many of the studies evaluated.

Concerns about *Campylobacter* spp. BSI relapses in patients with PIDs have been addressed previously. In a series of 68 *Campylobacter* spp. BSI, Fernández-Cruz et al. [34], reported three patients with recurrent *C. jejuni*, all of them suffering with humoral immunity deficiency. Moreover, in a paediatric study [10], all relapses occurred in patients with common variable immunodeficiency or agammaglobulinemia, even months or years after the initial episode. The cause of the high risk of relapse in this population is unknown. In our single-case description, the proportion of *C. coli* BSI relapses was two times that of *C. jejuni* and four-times that of *C. fetus*. The same was observed in other published studies [18, 50–52]. However, this is likely related to reporting bias from case report studies. In fact, this observation could not be confirmed in our meta-analysis of primary research studies, which, on the contrary, and based on meta-regression analysis, actually suggested a lower rate of relapse among studies including a higher proportion of participants with *C. jejuni*, and no significant relationship between relapse and the proportion of cases with *C. fetus* or *C.coli* included in the studies.

One possible explanation for the increases risk of relapse in hypogammaglobulinemia might be the insufficient levels of *Campylobacter* specific immunoglobulins in commercial substitutive immunoglobulins preparations, as previously described for *C. fetus* [53]. The concomitant deficit of other immunoglobulins important in controlling mucosal infections, such as immunoglobulin A, or the persistence of an intestinal reservoir of *Campylobacter* spp. might be other possible explanations [54]. The more frequent presentation with diarrhea and the higher rate of fecal isolation of *C. coli* and *C. jejuni* BSIs, as observed in individual case analyses, may suggest persistence within the gut microbiota. A recent study [6] comparing patients with common variable immunodeficiency with and without *Campylobacter* spp. infection (including BSI cases) showed that those with any *Campylobacter* spp. infection were more severely immunosuppressed, with lower counts of CD4^+^ and CD8^+^ T-cells, B-cells, and NK-cells over time.

The lack of established antimicrobial therapy recommendations for *Campylobacter* spp. BSI, especially in PID patients, poses serious problems for clinical management. In the case of the present systematic review, most of the included studies focused on clinical characteristics, *Campylobacter* spp. and resistance profiles, while many studies lacked a description of the treatment administered. Furthermore, many of the studies did not report outcomes according to the prescribed antimicrobial regimes. The high heterogeneity of the antimicrobial used also precluded us from drawing conclusions about the optimal antimicrobial therapy for avoiding mortality and/or relapses. However, some aspects might be considered. First, the high rates of quinolone resistance described may discourage its use as an empirical therapy [6, 9, 12, 15]. Second, in light of the results of the systematic review of case reports and short case series, macrolides should also be avoided as an empirical therapy for patients with PIDs.

The present study has several limitations which should be considered. Firstly, the high heterogeneity of the studies included and the lack of control groups may limit the generalizability of the results. Second, many studies did not report important variables such as the presence of PIDs, relapse rate or response to antimicrobial therapy, preventing risk factors analyses. Finally, individual case reports and small case series are likely affected by publication/reporting bias, where cases of particular phenotypes, more severe cases and/or cases with unusual/atypical evolution are published, and this likely explain, at least in part, some differences with the findings of primary research studies, such as the difference in the frequencies of *Campylobacter* spp. or the incidence of extravascular infections.

Despite these limitations, this systematic review and meta-analysis shows that *Campylobacter* spp. BSI poses a significant clinical challenge, particularly in immunocompromised populations. The high antimicrobial resistance rates, mortality and risk of relapse, highlight the need for implementing appropriate and evidence-based management strategies. This study also provides a basis for future research to address the gaps and inconclusive evidence observed. Large, well-designed prospective cohorts and clinical trials are needed to identify the optimal antimicrobial strategy and establish risk factors for antibiotic resistance, mortality and relapses. Specifically, studies focusing on PID populations are critical, given the condition’s high relapse rate.

## Supplementary Information

Below is the link to the electronic supplementary material.

Supplementary Material 1 (DOCX 426 KB)

## Data Availability

Data sharing not applicable as the present work relied on already published aggregated data, and all data collected and generated for this study are published in the present publication.

## References

[1] The European Union One Health 2022 Zoonoses Report [Internet]. [cited 20 september 2024]. Available at: https://www.efsa.europa.eu/en/efsajournal/pub/8442

[2] Trends in foodborne illness in the United States (2012) Atlanta: Centers for Disease Control and Prevention, April 18, 2013 (http://www.cdc.gov/features/

[3] Nielsen H, Hansen KK, Gradel KO, Kristensen B, Ejlertsen T, Østergaard C et al (2010) Bacteraemia as a result of *Campylobacter* species: a population-based study of epidemiology and clinical risk factors. Clin Microbiol Infect 16(1):57–61. 10.1111/j.1469-0691.2009.02900.x 19673969 10.1111/j.1469-0691.2009.02900.x

[4] Moffatt CRM, Kennedy KJ, O’Neill B, Selvey L, Kirk MD (2021) Bacteraemia, antimicrobial susceptibility and treatment among *Campylobacter*-associated hospitalisations in the Australian Capital Territory: a review. BMC Infect Dis 21(1):848. 10.1186/s12879-021-06558-x 34419003 10.1186/s12879-021-06558-x PMC8379883

[5] Tau L, Adler A, Shalom O, Paran Y, Poradosu RC, Shasha D et al (2022) Clinical features and outcomes of bacteremic and non-bacteremic campylobacteriosis: a case–control study. Infection 50(5):1225–31. 10.1007/s15010-022-01798-8 35316528 10.1007/s15010-022-01798-8

[6] Roa-Bautista A, Brown LAK, Tadros S, Burns SO, Godbole G, Lowe DM (2023) Clinical features, immunological characteristics, and treatment outcomes of *Campylobacter* spp. infections in patients with common variable immunodeficiency. J Allergy Clin Immunol Pract 11(11):3493-3501e4. 10.1016/j.jaip.2023.06.050 37406804 10.1016/j.jaip.2023.06.050

[7] Gazaigne L, Legrand P, Renaud B, Bourra B, Taillandier E, Brun-Buisson C et al (2008) *Campylobacter fetus* bloodstream infection: risk factors and clinical features. Eur J Clin Microbiol Infect Dis 27(3):185–9. 10.1007/s10096-007-0415-0 17999095 10.1007/s10096-007-0415-0

[8] Hussein K, Raz-Pasteur A, Shachor-Meyouhas Y, Geffen Y, Oren I, Paul M et al (2016) *Campylobacter* bacteraemia: 16 years of experience in a single centre. Infect Dis 48(11–12):796–9. 10.1080/23744235.2016.1195916 10.1080/23744235.2016.1195916 27320494

[9] Liao CH, Chuang CY, Huang YT, Lee PI, Hsueh PR (2012) Bacteremia caused by antimicrobial resistant *Campylobacter* species at a medical center in Taiwan, 1998–2008. J Infect 65(5):392–9. 10.1016/j.jinf.2012.06.014 22771419 10.1016/j.jinf.2012.06.014

[10] Ben-Shimol S, Carmi A, Greenberg D (2013) Demographic and clinical characteristics of *Campylobacter* bacteremia in children with and without predisposing factors. Pediatr Infect Dis J 32(11):e414–e418. 10.1097/INF.0b013e31829baae0 23694835 10.1097/INF.0b013e31829baae0

[11] Jirapongsananuruk O, Wanotayan K, Phongsamart W, Chokephaibulkit K, Visitsunthorn N, Luangwedchakarn V et al (2006) Recurrent *Campylobacter lari* bacteremia in X-linked agammaglobulinemia: a case report and review. Asian Pac J Allergy Immunol 24(2–3):171–174 17136884

[12] Tinévez C, Velardo F, Ranc AG, Dubois D, Pailhoriès H, Codde C et al (2022) Retrospective Multicentric Study on *Campylobacter* spp. Bacteremia in France: The Campylobacteremia Study. Clin Infect Dis 75(4):702–709. 10.1093/cid/ciab983 34849656 10.1093/cid/ciab983

[13] Pacanowski J, Lalande V, Lacombe K, Boudraa C, Lesprit P, Legrand P et al (2008) *Campylobacter* bacteremia: clinical features and factors associated with fatal outcome. Clin Infect Dis 47(6):790–6. 10.1086/591530 18699745 10.1086/591530

[14] Tinévez C, Lehours P, Ranc AG, Belaroussi Y, Cazanave C, Puges M et al (2023) Multicenter retrospective study of vascular infections and endocarditis caused by *Campylobacter* spp., France. Emerg Infect Dis 29(3):484–92. 10.3201/eid2903.221417 36823023 10.3201/eid2903.221417 PMC9973684

[15] O’Hara GA, Fitchett JRA, Klein JL (2017) *Campylobacter* bacteremia in London: a 44-year single-center study. Diagn Microbiol Infect Dis 89(1):67–71. 10.1016/j.diagmicrobio.2017.05.015 28629878 10.1016/j.diagmicrobio.2017.05.015

[16] Feodoroff B, Lauhio A, Ellstrom P, Rautelin H (2011) A nationwide study of *Campylobacter jejuni* and *Campylobacter coli* bacteremia in Finland over a 10-year period, 1998–2007, with special reference to clinical characteristics and antimicrobial susceptibility. Clin Infect Dis 53(8):e99-106. 10.1093/cid/cir509 21921217 10.1093/cid/cir509 PMC3174097

[17] Sunnerhagen T, Grenthe R, Kampmann C, Karlsson Söbirk S, Bläckberg A (2024) *Campylobacter* infections with and without bacteremia: a comparative retrospective population-based study. Open Forum Infect Dis 11(3):ofae131. 10.1093/ofid/ofae131 38524227 10.1093/ofid/ofae131 PMC10960602

[18] Tokuda K, Nishi J, Miyanohara H, Sarantuya J, Iwashita M, Kamenosono A et al (2004) Relapsing cellulitis associated with *Campylobacter coli* bacteremia in an agammaglobulinemic patient. Pediatr Infect Dis J 23(6):577–9. 10.1097/01.inf.0000130080.86862.d5 15194845 10.1097/01.inf.0000130080.86862.d5

[19] Arai A, Kitano A, Sawabe E, Kanegane H, Miyawaki T, Miura O (2007) Relapsing *Campylobacter coli* bacteremia with reactive arthritis in a patient with X-linked agammaglobulinemia. Intern Med 46(9):605–9. 10.2169/internalmedicine.46.6108 17473499 10.2169/internalmedicine.46.6108

[20] European Society for Immunodeficiencies [Internet]. ESID (2023) [cited 05 july 2024]. Available at: https://esid.org/Education/Diagnostic-Criteria-PID

[21] Page MJ, McKenzie JE, Bossuyt PM, Boutron I, Hoffmann TC, Mulrow CD et al (2021) The PRISMA 2020 statement: an updated guideline for reporting systematic reviews. BMJ n71. 10.1136/bmj.n71 10.1136/bmj.n71 PMC8005924 33782057

[22] Meda S, Shenoy P, Kumar G, Varma M, Mukhopadhyay C, Kalwaje Eshwara V (2024) Case report: *Campylobacter* bacteremia in India. Am J Trop Med Hyg 111(5):1066–1069. 10.4269/ajtmh.24-0193 39191243 10.4269/ajtmh.24-0193 PMC11542528

[23] Baek YJ, Song JE, Kim EJ, Choi H, Sohn Y, Jeon YD et al (2024) Trends, clinical characteristics, antimicrobial susceptibility patterns, and outcomes of *Campylobacter* bacteraemia: a multicentre retrospective study. Infection 52(3):857–64. 10.1007/s15010-023-02118-4 37910310 10.1007/s15010-023-02118-4

[24] Graham A, Hawkins L, Balasegaram S, Narasimhan S, Wain J, Clarke J et al (2024) A decade of *Campylobacter* and *Campylobacter* bacteraemias in a district general hospital and the surrounding London and South East region, England. J Infect 88(1):15–20. 10.1016/j.jinf.2023.11.004 37995801 10.1016/j.jinf.2023.11.004

[25] Zayet S, Klopfenstein T, Gendrin V, Vuillemenot JB, Plantin J, Toko L et al (2023) *Campylobacter fetus* invasive infections and risks for death, France, 2000–2021. Emerg Infect Dis 29(11):2189–97. 10.3201/eid2911.230598 37877803 10.3201/eid2911.230598 PMC10617355

[26] Otsuka Y, Hagiya H, Takahashi M, Fukushima S, Maeda R, Sunada N et al (2023) Clinical characteristics of *Campylobacter* bacteremia: a multicenter retrospective study. Sci Rep 13(1):647. 10.1038/s41598-022-27330-4 36635328 10.1038/s41598-022-27330-4 PMC9837072

[27] Shahunja KM, Ahmed T, Hossain MI, Islam MM, Monjory MB, Shahid ASMSB et al (2020) Clinical and laboratory characteristics of children under five hospitalized with diarrhea and bacteremia. PLoS One 15(12):e0243128. 10.1371/journal.pone.0243128 33264364 10.1371/journal.pone.0243128 PMC7710075

[28] Liu YH, Yamazaki W, Huang YT, Liao CH, Sheng WH, Hsueh PR (2019) Clinical and microbiological characteristics of patients with bacteremia caused by *Campylobacter* species with an emphasis on the subspecies of *C. fetus*. J Microbiol Immunol Infect 52(1):122–31. 10.1016/j.jmii.2017.07.009 28801089 10.1016/j.jmii.2017.07.009

[29] Yamamoto K, Hayakawa K, Nagashima M, Shimada K, Kutsuna S, Takeshita N et al (2017) Comparison of the clinical and microbiological characteristics of *Campylobacter* and *Helicobacter* bacteremia: the importance of time to blood culture positivity using the BACTEC blood culture systems. BMC Res Notes 10(1):634. 10.1186/s13104-017-2981-2 29183353 10.1186/s13104-017-2981-2 PMC5704506

[30] Marchand-Senécal X, Bekal S, Pilon PA, Sylvestre JL, Gaudreau C (2017) *Campylobacter fetus* cluster among men who have sex with men, Montreal, Quebec, Canada, 2014–2016. Clin Infect Dis 65(10):1751–3. 10.1093/cid/cix610 29020280 10.1093/cid/cix610

[31] Cypierre A, Denes E, Barraud O, Jamilloux Y, Jacques J, Durox H et al (2014) *Campylobacter fetus* infections. Med Mal Infect 44(4):167–73. 10.1016/j.medmal.2014.02.001 24637053 10.1016/j.medmal.2014.02.001

[32] Mori T, Hasegawa N, Sugita K, Shinjoh M, Nakamoto N, Shimizu T et al (2014) Clinical features of bacteremia due to *Campylobacter jejuni*. Intern Med 53(17):1941–4. 10.2169/internalmedicine.53.2559 25175126 10.2169/internalmedicine.53.2559

[33] Fica CA, Porte TL, Braun JS, Veas PN, Pavez AC, Dabanch PJ et al (2011) Bacteriemias e infección endovascular por *Campylobacter* spp: nuestra experiencia en un cuarto de siglo de historia. Rev Chilena Infectol 28(3):211–6 21879145

[34] Fernández-Cruz A, Muñoz P, Mohedano R, Valerio M, Marín M, Alcalá L et al (2010) *Campylobacter* bacteremia: clinical characteristics, incidence, and outcome over 23 years. Medicine (Baltimore) 89(5):319–30. 10.1097/MD.0b013e3181f2638d 20827109 10.1097/MD.0b013e3181f2638d

[35] Cochennec F, Gazaigne L, Lesprit P, Desgranges P, Allaire E, Becquemin JP (2008) Aortoiliac aneurysms infected by *Campylobacter fetus*. J Vasc Surg 48(4):815–20. 10.1016/j.jvs.2008.05.076 18692356 10.1016/j.jvs.2008.05.076

[36] Woo PCY, Leung KW, Tsoi HW, Wong SSY, Teng JLL, Yuen KY (2002) Thermo-tolerant *Campylobacter fetus* bacteraemia identified by 16S ribosomal RNA gene sequencing: an emerging pathogen in immunocompromised patients. J Med Microbiol 51(9):740–6. 10.1099/0022-1317-51-9-740 12358064 10.1099/0022-1317-51-9-740

[37] Tee W, Mijch A (1998) *Campylobacter jejuni* bacteremia in human immunodeficiency virus (HIV)- infected and non‐HIV‐infected patients: comparison of clinical features and review. Clin Infect Dis 26(1):91–6. 10.1086/516263 9455515 10.1086/516263

[38] Pigrau C, Bartolome R, Almirante B, Planes A, Gavalda J, Pahissa A (1997) Bacteremia due to *Campylobacter* species: clinical findings and antimicrobial susceptibility patterns. Clin Infect Dis 25(6):1414–1420. 10.1086/516127 9431389 10.1086/516127

[39] Font C, Cruceta A, Moreno A, Miró O, Coll-Vinent B, Almela M et al (1997) A study of 30 patients with bacteremia due to *Campylobacter* spp. Med Clin (Barc) 108(9):336–340 9139156

[40] Pigrau C, Almirante B, Pahissa A, Bartolomé R (1996) *Campylobacter* spp. bacteremia in AIDS patients. J Acquir Immune Defic Syndr Hum Retrovirol 12(1):93. 10.1097/00042560-199605010-00013 8624767 10.1097/00042560-199605010-00013

[41] Schønheyder HC, Søgaard P, Frederiksen W (1995) A survey of *Campylobacter* bacteremia in three Danish counties, 1989 to 1994. Scand J Infect Dis 27(2):145–8. 10.3109/00365549509018995 7660078 10.3109/00365549509018995

[42] Skirrow MB, Jones DM, Sutcliffe E, Benjamin J (1993) *Campylobacter* bacteraemia in England and Wales, 1981-91. Epidemiol Infect 110(3):567–73. 10.1017/s0950268800050986 8519321 10.1017/s0950268800050986 PMC2272297

[43] NIH Quality Assessment Tool for Case Series Studies [Internet] Available at: https://www.nhlbi.nih.gov/health-topics/study-quality-assessment-tools

[44] Posit team, R Core Team (2023) RStudio: Integrated Development Environment for R. Posit Software, PBC, Boston, MA. URL http://www.posit.co/. (2022). R: A language and environment for statistical computing. R Foundation for Statistical Computing, Vienna, Austria. URL https://www.R-project.org/

[45] Balduzzi S, Rücker G, Schwarzer G (2019) How to perform a meta-analysis with R: a practical tutorial. Evid Based Ment Health 22(4):153–160. 10.1136/ebmental-2019-300117 31563865 10.1136/ebmental-2019-300117 PMC10231495

[46] Cardenas-Morales M, Hernandez-Trujillo VP (2021) Agammaglobulinemia: from X-linked to autosomal forms of disease. Clinic Rev Allerg Immunol 63(1):22–35. 10.1007/s12016-021-08870-5 10.1007/s12016-021-08870-5 PMC8269404 34241796

[47] Obata S, Mochida Y, Hidaka S, Kobayashi S (2023) Cardiac tamponade secondary to *Campylobacter fetus* infection in a kidney transplant recipient. J Am Soc Nephrol 34(11S):618–9. 10.1681/ASN.20233411S1618d

[48] Lenherr A, Boughdad S, Prior JO, Lalonde MN, Filippidis P (2024) Infective aortitis and subacute myocarditis due to *Campylobacter fetus*. Int J Infect Dis 138:113–4. 10.1016/j.ijid.2023.11.033 38016501 10.1016/j.ijid.2023.11.033

[49] Najjar I, Paluca F, Loukidis K, Tarr PE (2020) Recurrent *Campylobacter* enteritis in patients with hypogammaglobulinemia: review of the literature. J Clin Med 9(2):553. 10.3390/jcm9020553 32085573 10.3390/jcm9020553 PMC7074135

[50] Jiang L, Gao J, Wang P, Liu Y (2022) Relapsing cellulitis associated with *Campylobacter* coli bacteremia in a Good’s syndrome patient: a case report. BMC Infect Dis 22(1):354. 10.1186/s12879-022-07324-3 35397507 10.1186/s12879-022-07324-3 PMC8994272

[51] Bonilla-Moreno M, Torrecillas M, Laporte-Amargos J, González-Díaz A, Mussetti A, Tubau F et al (2023) Development of meropenem resistance in a multidrug-resistant *Campylobacter coli* strain causing recurrent bacteremia in a hematological malignancy patient. Antimicrob Agents Chemother. 10.1128/aac.00272-23 37358413 10.1128/aac.00272-23 PMC10648861

[52] Hagiya H, Kimura K, Nishi I, Yoshida H, Yamamoto N, Akeda Y et al (2018) Emergence of carbapenem non-susceptible *Campylobacter coli* after long-term treatment against recurrent bacteremia in a patient with X-linked agammaglobulinemia. Intern Med 57(14):2077–80. 10.2169/internalmedicine.0312-17 29491300 10.2169/internalmedicine.0312-17 PMC6096023

[53] Neuzil KM, Wang E, Haas DW, Blaser MJ (1994) Persistence of *Campylobacter fetus* bacteremia associated with absence of opsonizing antibodies. J Clin Microbiol 32(7):1718–20. 10.1128/jcm.32.7.1718-1720.1994 7929763 10.1128/jcm.32.7.1718-1720.1994 PMC263773

[54] Okada H, Kitazawa T, Harada S, Itoyama S, Hatakeyama S, Ota Y et al (2008) Combined treatment with oral kanamycin and parenteral antibiotics for a case of persistent bacteremia and intestinal carriage with *Campylobacter coli*. Intern Med 47(14):1363–6. 10.2169/internalmedicine.47.1161 18628588 10.2169/internalmedicine.47.1161

