# Supplementary material for: The clinical characteristics and outcomes of Campylobacter spp. bloodstream infection: A systematic review and meta-analysis
Source: Eur J Clin Microbiol Infect Dis. 2026 Mar 13;45(6):1541–56. doi: 10.1007/s10096-026-05465-w (PMC13319890; doi:10.1007/s10096-026-05465-w)
Supplement: Supplementary file 1 — Supplementary Material 1 (DOCX 426 KB) [file 10096_2026_5465_MOESM1_ESM.docx]

**Clinical characteristics and outcomes of *Campylobacter*spp. bloodstream infection: A systematic review and meta-analysis.**

**SUPPLEMENTARY MATERIAL**

Supplemental Tables S1 to S11.

References of individual case series and case reports included in the systematic review

Supplemental Figures 1 to 3

**Supplemental Table S1.** The completed PRISMA checklist (13)

| **Section and Topic** | **Item #** | **Checklist item** | **Location where item is reported** |
| --- | --- | --- | --- |
| **TITLE** | | | |
| Title | 1 | Identify the report as a systematic review. | 1 |
| **ABSTRACT** | | | |
| Abstract | 2 | See the PRISMA 2020 for Abstracts checklist. | 3 |
| **INTRODUCTION** | | | |
| Rationale | 3 | Describe the rationale for the review in the context of existing knowledge. | 5 |
| Objectives | 4 | Provide an explicit statement of the objective(s) or question(s) the review addresses. | 6 |
| **METHODS** | | | |
| Eligibility criteria | 5 | Specify the inclusion and exclusion criteria for the review and how studies were grouped for the syntheses. | 7 |
| Information sources | 6 | Specify all databases, registers, websites, organizations, reference lists and other sources searched or consulted to identify studies. Specify the date when each source was last searched or consulted. | 7 |
| Search strategy | 7 | Present the full search strategies for all databases, registers and websites, including any filters and limits used. | 7 |
| Selection process | 8 | Specify the methods used to decide whether a study met the inclusion criteria of the review, including how many reviewers screened each record and each report retrieved, whether they worked independently, and if applicable, details of automation tools used in the process. | 7 |
| Data collection process | 9 | Specify the methods used to collect data from reports, including how many reviewers collected data from each report, whether they worked independently, any processes for obtaining or confirming data from study investigators, and if applicable, details of automation tools used in the process. | 8 |
| Data items | 10a | List and define all outcomes for which data were sought. Specify whether all results that were compatible with each outcome domain in each study were sought (e.g. for all measures, time points, analyses), and if not, the methods used to decide which results to collect. | 8 |
|  | 10b | List and define all other variables for which data were sought (e.g. participant and intervention characteristics, funding sources). Describe any assumptions made about any missing or unclear information. | 8 |
| Study risk of bias assessment | 11 | Specify the methods used to assess risk of bias in the included studies, including details of the tool(s) used, how many reviewers assessed each study and whether they worked independently, and if applicable, details of automation tools used in the process. | 8 |
| Effect measures | 12 | Specify for each outcome the effect measure(s) (e.g. risk ratio, mean difference) used in the synthesis or presentation of results. | 8,9 |
| Synthesis methods | 13a | Describe the processes used to decide which studies were eligible for each synthesis (e.g. tabulating the study intervention characteristics and comparing against the planned groups for each synthesis (item #5)). | 8 |
|  | 13b | Describe any methods required to prepare the data for presentation or synthesis, such as handling of missing summary statistics, or data conversions. | 8 |
|  | 13c | Describe any methods used to tabulate or visually display results of individual studies and syntheses. | 8 |
|  | 13d | Describe any methods used to synthesize results and provide a rationale for the choice(s). If meta-analysis was performed, describe the model(s), method(s) to identify the presence and extent of statistical heterogeneity, and software package(s) used. | 8 |
|  | 13e | Describe any methods used to explore possible causes of heterogeneity among study results (e.g. subgroup analysis, meta-regression). | 8 |
|  | 13f | Describe any sensitivity analyses conducted to assess robustness of the synthesized results. | 8 |
| Reporting bias assessment | 14 | Describe any methods used to assess risk of bias due to missing results in a synthesis (arising from reporting biases). | 8,9 |
| Certainty assessment | 15 | Describe any methods used to assess certainty (or confidence) in the body of evidence for an outcome. | 8,9 |
| **RESULTS** | | | |
| Study selection | 16a | Describe the results of the search and selection process, from the number of records identified in the search to the number of studies included in the review, ideally using a flow diagram. | 10,11 |
|  | 16b | Cite studies that might appear to meet the inclusion criteria, but which were excluded, and explain why they were excluded. | NA |
| Study characteristics | 17 | Cite each included study and present its characteristics. | 10,11 |
| Risk of bias in studies | 18 | Present assessments of risk of bias for each included study. | 11, 12, S3 |
| Results of individual studies | 19 | For all outcomes, present, for each study: (a) summary statistics for each group (where appropriate) and (b) an effect estimate and its precision (e.g. confidence/credible interval), ideally using structured tables or plots. | 10, 11, 12, 13,14. 15, 16 |
| Results of syntheses | 20a | For each synthesis, briefly summarise the characteristics and risk of bias among contributing studies. | 12, 13,14,15, 16 |
|  | 20b | Present results of all statistical syntheses conducted. If meta-analysis was done, present for each the summary estimate and its precision (e.g. confidence/credible interval) and measures of statistical heterogeneity. If comparing groups, describe the direction of the effect. | 12, 13,14,15, 16 |
|  | 20c | Present results of all investigations of possible causes of heterogeneity among study results. | 12,13 |
|  | 20d | Present results of all sensitivity analyses conducted to assess the robustness of the synthesized results. | 12,13 |
| Reporting biases | 21 | Present assessments of risk of bias due to missing results (arising from reporting biases) for each synthesis assessed. | 12 |
| Certainty of evidence | 22 | Present assessments of certainty (or confidence) in the body of evidence for each outcome assessed. | 12 |
| **DISCUSSION** | | | |
| Discussion | 23a | Provide a general interpretation of the results in the context of other evidence. | 28, 29, 30, 31, 32 |
|  | 23b | Discuss any limitations of the evidence included in the review. | 28, 29, 30, 31, 32 |
|  | 23c | Discuss any limitations of the review processes used. | 28, 29, 30, 31, 32 |
|  | 23d | Discuss implications of the results for practice, policy, and future research. | 28, 29, 30, 31, 32 |
| **OTHER INFORMATION** | | | |
| Registration and protocol | 24a | Provide registration information for the review, including register name and registration number, or state that the review was not registered. | 6,7 |
|  | 24b | Indicate where the review protocol can be accessed, or state that a protocol was not prepared. | 6,7 |
|  | 24c | Describe and explain any amendments to information provided at registration or in the protocol. | NA |
| Support | 25 | Describe sources of financial or non-financial support for the review, and the role of the funders or sponsors in the review. | 38 |
| Competing interests | 26 | Declare any competing interests of review authors. | 38 |
| Availability of data, code and other materials | 27 | Report which of the following are publicly available and where they can be found: template data collection forms; data extracted from included studies; data used for all analyses; analytic code; any other materials used in the review. | 39 |

**Supplemental Table S2.** Full search strategy

| PubMed  Cochrane | (Campylobacter OR "Campylobacter"[Mesh] OR "Campylobacter upsaliensis"[Mesh] OR "Campylobacter sputorum"[Mesh] OR "Campylobacter rectus"[Mesh] OR "Campylobacter lari"[Mesh] OR "Campylobacter hyointes;nalis"[Mesh] OR "Campylobacter jejuni"[Mesh] OR "Campylobacter coli"[Mesh] OR "Campylobacter Infectons"[Mesh] OR "Campylobacter fetus"[Mesh] OR "Helicobacter mustelae"[Mesh]) AND ("Bacteremia"[Mesh] OR "Sepsis"[Mesh] OR Bacteremia OR sepsis OR sep;cemia OR bloodstream). |
| --- | --- |
| Embase | (campylobacter:ab,ti OR 'campylobacter jejuni':ab,ti) AND (bacteremia:ab,ti OR 'bloodstream infection':ab,ti) |

**Supplemental Table S3.** Grading the quality of evidence of the primary research studies (NIH Quality Assessment Tool for Case Series Studies).

| **Study** | **1.Was the study question or objective clearly stated?** | **2.Was the study population clearly and fully described, including a case definition?** | **3. Were the cases consecutive?** | **4. Were the subjects comparable?** | **5. Was the intervention clearly described?** | **6. Were the outcome measures clearly defined, valid, reliable, and implemented consistently across all study participants?** | **7. Was the length of follow-up adequate?** | **8. Were the statistical methods well-described?** | **9. Were the results well-described?** |
| --- | --- | --- | --- | --- | --- | --- | --- | --- | --- |
| Sunnerhagen et al, 2024 ^17^ | Yes | Yes | Yes | Yes | Yes | Yes | Yes | Yes | Yes |
| Meda et al, 2024 ^36^ | Yes | Yes | Yes | NA | Yes | Yes | Yes | Yes | Yes |
| Baek et al, 2024 ^37^ | Yes | Yes | No | NA | Yes | Yes | Yes | Yes | Yes |
| Graham, 2024 ^38^ | Yes | Yes | No | No | Yes | Yes | Yes | Yes | Yes |
| Zayet et al, 2023 ^25^ | Yes | Yes | NA | Yes | Yes | Yes | NA | Yes | Yes |
| Otsuka et al., 2023 ^33^ | Yes | Yes | Yes | No | NA | Yes | Yes | Yes | Yes |
| Tinévez et al., 2023 ^14^ | Yes | Yes | Yes | No | NA | Yes | Yes | Yes | Yes |
| Tinévez et al., 2022 ^12^ | Yes | Yes | Yes | No | NA | Yes | Yes | Yes | Yes |
| Tau et al., 2022 ^5^ | Yes | Yes | Yes | Yes | NA | Yes | Yes | Yes | Yes |
| Shahunja et al., 2020 ^39^ | Yes | Yes | Yes | Yes | NA | Yes | Yes | Yes | Yes |
| Liu et al., 2019 ^40^ | Yes | Yes | Yes | No | NA | Yes | Yes | Yes | Yes |
| O'Hara et al, 2017 ^15^ | Yes | Yes | Yes | Yes | NA | NA | Yes | Yes | Yes |
| Yamamoto et al, 2017 ^34^ | Yes | Yes | Yes | No | NA | NA | Yes | Yes | Yes |
| Marchand- Senécal et al., 2017 ^41^ | Yes | Yes | Yes | Yes | NA | NA | Yes | Yes | Yes |
| Cypierre et al., 2014 ^26^ | Yes | Yes | Yes | Yes | NA | NA | Yes | Yes | Yes |
| Mori et al., 2014 ^32^ | Yes | Yes | No | NA | NA | NA | Yes | NA | Yes |
| Ben-Shimol et al., 2013 ^10^ | Yes | Yes | No | NA | NA | NA | Yes | NA | Yes |
| Liao et al., 2012 ^9^ | Yes | Yes | Yes | No | NA | NA | NA | No | Yes |
| Feodoroff et al., 2011 ^16^ | Yes | Yes | Yes | Yes | NA | NA | Yes | NA | Yes |
| Fica et al., 2011 ^42^ | Yes | Yes | No | No | NA | NA | NA | NA | Yes |
| Nielsen et al., 2010 ^3^ | Yes | Yes | No | Yes | NA | NA | NA | Yes | Yes |
| Fernández-Cruz et al., 2010 ^29^ | Yes | Yes | No | Yes | NA | NA | NA | Yes | Yes |
| Cochennec et al.,2008 ^27^ | Yes | Yes | Yes | Yes | NA | NA | NA | NA | Yes |
| Gazaigne et al., 2008 ^7^ | Yes | Yes | Yes | No | NA | Yes | Yes | Yes | Yes |
| Pacanowski et al., 2008 ^13^ | Yes | Yes | Yes | Yes | NA | NA | NA | Yes | Yes |
| Sunnerhagen et al, 2024 ^17^ | Yes | Yes | Yes | Yes | NA | NA | NA | NA | Yes |
| Meda et al, 2024 ^36^ | Yes | Yes | Yes | Yes | NA | Yes | Yes | Yes | Yes |
| Baek et al, 2024 ^37^ | Yes | Yes | Yes | Yes | NA | Yes | Yes | Yes | Yes |
| Graham, 2024 ^38^ | Yes | Yes | NA | Yes | NA | Yes | Yes | Yes | Yes |
| Zayet et al, 2023 ^25^ | Yes | Yes | NA | Yes | Yes | Yes | Yes | Yes | Yes |
| Otsuka et al., 2023 ^33^ | Yes | Yes | NA | Yes | Yes | Yes | Yes | Yes | Yes |
| Tinévez et al., 2023 ^14^ | Yes | Yes | No | NA | NA | NA | NA | NA | Yes |

NA: not applicable.

**Supplemental Table S4.** Baseline characteristics and comorbidities of patients with *Campylobacter* spp. BSI included in 32 original research articles.

| **Study** | **n** | **Sex (male)**  **n (%)** | **Age (years)** | **N patients included in mortality or relapse meta-analyses** | **Overall (N) prevalence of comorbidities*** | **Comorbidity types** | | | | | | | | | | |
| --- | --- | --- | --- | --- | --- | --- | --- | --- | --- | --- | --- | --- | --- | --- | --- | --- |
|  |  |  |  |  |  | **Immunocompromise**  **n (%)** | **Solid**  **neoplasm**  **n (%)** | **Haematological malignancy**  **n (%)** | **SOT**  **n (%)** | **HSCT**  **n (%)** | **IS therapy**  **n (%)** | **Other IS**  **n (%)** | **HIV**  **n (%)** | **Alcohol**  **n (%)** | **Diabetes**  **n (%)** | **Liver**  **disease**  **n (%)** |
| Sunnerhagen et al, 2024 (17) | 29 | 22 (76) | 72 (median, IQR 56-82) | MM: 29  RM: 0 | 9 | 5 (17) | NR | NR | NR | NR | NR | NR | NR | NR | 3 (10) | 1 (3) |
| Meda et al, 2024 (44) | 12 | 9 (75) | 55.5 (median, IQR 42.5–58.75) | MM: 12  RM: 0 | 16 | 1 (8.3) | 1 (8.3) | 0 | 0 | 0 | NR | NR | 3 (25) | NR | 3 (25) | 8 (75) |
| Baek et al, 2024 (45) | 108 | 78 (72) | 59 (median, IQR 37–72) | MM: 108  RM: 0 | 84 | NR | 23 (23.3) | 8 (7.4) | 3 (2.8) | NR | NR | 12 (11.1) | 2 (1.9) | NR | 27 (25) | 9 (8.7) |
| Graham, 2024 (35) | 34 | 21 (62) | 75 (median, IQR 53 – 80) | MM: 34  RM: 34 | 39 | 25 (73.5) | 5 (14.7) | 2 (5.9) | NR | NR | NR | NR | NR | NR | 7 (21.2) | NR |
| Zayet et al, 2023 (25) | 21 | 12 (57) | 73 (mean, SD 18) | MM: 21  RM: 21 | 26 | 11 (52) | 7 (33) | 7 (33) | 0 | NR | NR | NR | 0 | NR | 7 (33) | 1 (4.8) |
| Otsuka et al., 2023 (33) | 39 | 24 (61.5) | 57 (median, IQR 28-75) | MM: 39  RM: 0 | 37 | NR | 5 (12.8) | 8 (20.5) | NR | NR | 6 (15.4) | 5 (12.8) | NR | NR | 8 (20.5) | 5 (12.8) |
| Tinévez et al., 2023 (14) | 57 | 36 (80) | 69.5 (median, IQR 61–81) | MM: 0  RM: 0 | 36 | 7 (15.6) | 11 (24.4) | 2 (4.4) | NR | NR | NR | NR | NR | NR | 11 (24.4) | 5 (11.1) |
| Tinévez et al., 2022 (12) | 592 | 402 (67.9) | 68 (median, IQR 53–78) | MM: 592  RM: 592 | 1983 | 257 (43.4) | 135 (23) | 151 (25.9) | 26 (4.4) | 20 (3.4) | 176 (30.2) | 112 (19.3) | 3 (0.5) | NR | 128 (22.3) | 75 (12.3) |
| Tau et al., 2022 (5) | 76 | 48 (63.1) | 67 (median, IQR 38–82) | MM: 76  RM: 76 | 154 | 46 (60.5) | 10 (13.1) | 29 (38.1) | NR | NR | QT: 25 (32.8) | 25 (32.8) | NR | NR | 19 (25) | NR |
| Shahunja et al., 2020 (40) | 9 | NR | NR | MM: 9  RM: 0 | NR | NR | NR | NR | NR | NR | NR | NR | NR | NR | NR | NR |
| Liu et al., 2019 (38) | 56 | 35 (62.5) | 54 (mean, range 8-89) | MM: 56  RM: 0 | 51 | NR | 17 (30.4) | 9 (16.1) | 4 (7.1) | 1 (1.8) | NR | NR | NR | NR | 7 (12.5) | 13 (23.2) |
| O'Hara et al, 2017 (15) | 41 | 27 (66) | 46 (mean, SD ± 20.9) | MM: 41  RM: 0 | 17 | NR | 5 (12.2) | NR | NR | NR | NR | NR | 4 (9.8) | NR | 4 (9.8) | 4 (9.8) |
| Yamamoto et al, 2017 (34) | 14 | 8 (57) | 49 (mean, SD ± 21) | MM: 14  RM: 14 | 8 | NR | 4 (28.6) | 1 (7.1) | NR | NR | 1 (7.1) | NR | NR | NR | NR | 2 (14.2) |
| Marchand- Senécal et al., 2017 (39) | 3 | 3 (100) | 60 (median, IQR 56–67) | MM: 3  RM: 0 | 2 | NR | 1 (33.3) | NR | NR | NR | NR | NR | 1 (33.3) | NR | NR | NR |
| Cypierre et al., 2014 (26) | 20 | 15 (75) | 73 (mean, range 43-91) | MM: 29  RM: 0 | 35 | NR | 12 (60) | 9 (45) | NR | NR | 8 (40) | 3 (15) | NR | 2 (10) | 5 (25) | 1 (5) |
| Mori et al., 2014 (32) | 7 | 5 (71) | 54 (median, IQR 19-77) | MM: 7  RM: 7 | 7 | 2 (14.3) | NR | 2 (28.6) | NR | NR | NR | NR | NR | NR | NR | 3 (42.9) |
| Ben-Shimol et al., 2013 (10) | 60 | NR | <19 | MM: 60  RM: 0 | 13 | XLA 6 (10)  Splenectomy 2 (3.3) | NR | 4 (5) | NR | NR | NR | NR | NR | NR | NR | 1 (1.67) |
| Liao et al., 2012 (9) | 24 | 16 (67) | 45.9 (mean, range 7-83) | MM: 24  RM: 0 | 29 | NR | 8 (33) | NR | NR | NR | NR | 7 (29) | NR | NR | 5 (21) | 9 (38) |
| Feodoroff et al., 2011 (16) | 76 | 56 (74) | 46 (median, range 1–95) | MM: 76  RM: 0 | NR | NR | NR | NR | NR | NR | NR | NR | NR | NR | NR | NR |
| Fica et al., 2011 (42) | 6 | 4 (66.7) | 32.4 (mean, range 19-63) | MM: 6  RM: 0 | 2 | 0 | 0 | 1 (14.3) | 0 | 0 | 0 | 0 | 0 | NR | 0 | 1 (14.3) |
| Nielsen et al., 2010 (3) | 46 | 33 (72) | 56 (median, range 1–94) | MM: 46  RM: 46 | 13 | NR | NR | NR | NR | NR | 6 (12) | NR | 4 (8.7) | NR | 3 (6.5) | NR |
| Fernández-Cruz et al., 2010 (29) | 68 | 56  (82.4) | 52 (median, IQR 31.3 - 72.5) | MM: 68  RM: 68 | 81 | 33 (51.6) | 3 (4.7) | 4 (6.3) | 2 (3) | NR | NR | 3 (4.7) | 15 (23.4) | 4.7% | NR | 21 (32.8) |
| Cochennec et al.,2008 (27) | 5 | 5 (100) | 76 (mean, range 69-79) | MM: 0  RM: 0 | 4 | NR | 2 (40) | 1 (20) | NR | NR | NR | NR | NR | 1 (20) | NR | NR |
| Gazaigne et al., 2008 (7) | 21 | 18 (85.7) | 78 (median, range 30 -  95) | MM: 21  RM: 21 | 12 | NR | 1 (4.8) | NR | NR | NR | NR | 3 (14.3) | NR | NR | 5 (23.8) | 3 (14.3) |
| Pacanowski et al., 2008 (13) | 178 | 124 (70) | 64 (mean, range 6–97) | MM: 178  RM: 0 | 406 | 141 (79.2)  Neutropenia: 7.3%  Splenectomy: 2.2% | 46 (25.8) | 21 (12) | 6 (3) | NR | QT 43 (24) | 32 (18) | 17 (10) | NR | 31 (17) | 69 (39) |
| Woo et al.,2002 (41) | 8 | 4 (50) | 64 (median, range 51–82) | MM: 8  RM: 0 | 10 | NR | 1 (12.5) | 1 (12.5) | NR | NR | NR | NR | NR | 1 (12.5) | 2 (25) | 5 (62.5) |
| Tee et al., 1998 (43) | 21 | 20 (80.9) | NR | MM: 21  RM: 0 | 12 | NR | NR | NR | NR | NR | QT: 1 (4.2) | 1 (4.2) | 10 (41.7) | NR | NR | NR |
| Pigrau et al., 1997 (30) | 58 | 38 (66) | 39.4 (mean, range, 4 days - 87 years) | MM: 58  RM: 58 | 69 | NR | 8 (14) | 2 (3.45) | 5 (9) | NR | QT: 10 (17) | 13 (22) | 7 (12) | NR | 4 (7) | 20 (34) |
| Font et al., 1997 (28) | 30 | 22 (73) | 52 (mean, SD ± 19 | MM: 30  RM: 30 | 45 | Splenectomy: 1 (3.3) | 6 (40) | 4 (13.3) | 2 (6.7) | 1 (3.3) | 4 (13) | 2 (6.7) | 7 (23) | NR | 3 (10) | 15 (50) |
| Pigrau et al., 1996 (31) | 7 | 6 (86) | 24 (mean, range 8 months -31 years) | MM: 0  RM: 0 | 7 | NR | NR | NR | NR | NR | NR | NR | 7 (100) | NR | NR | NR |
| Schonheyder et al., 1995 (37) | 15 | 7 (46.7) | 42 (median, range 15-90) | MM: 15  RM: 15 | 10 | XLA: 1 (6.7) | 0 | NR | 1 (6.7) | NR | NR | 3 (42.9) | 1 (6.7) | 1 (6.7) | 2 (13.3) | 1 (6.7) |
| Skirrow et al., 1993 (36) | 394 | NR | NR | MM: 394  RM: 0 | 145 | 74 (29.6) | 30 (27.5) | NR | NR | NR | NR | NR | 11 (10.1) | NR | 15 (13.8) | 15 (13.8) |

*Patients may have more than one comorbidity.

MM: Mortality meta-analysis; RM: relapse meta-analysis; IS: immunosuppressant drugs; XLA: X-linked agammaglobulinemia; AIDS: acquired immune deficiency syndrome; HIV: human immunodeficiency virus; QT: chemotherapy; SOT: solid organ transplantation; HSCT: haematopoietic stem cell transplant; SD: standard deviation; IQR: interquartile range; NR: not reported.

**Supplemental Table S5.** Clinical and microbiological characteristics of *Campylobacter* spp. BSI included in 32 original research articles.

| **Study** | **n** | **N patients included in mortality or relapse meta-analyses** | **Fever**  **n (%)** | **Diarrhoea**  **n (%)** | **Abdominal pain**  **n (%)** | **Extra-intestinal manifestations**  **n (%)** | ***Campylobacter* spp.**  **n (%)** | ***Stool culture***  **n (%)** | **Campylobacter spp. resistance**  **n (%)** | ***C. col*i resistance**  **n (%)** | ***C. jejuni***  ***resistance***  **n (%)** | ***C. fetus***  ***resistance***  **n (%)** |
| --- | --- | --- | --- | --- | --- | --- | --- | --- | --- | --- | --- | --- |
| Sunnerhagen et al, 2024 (17) | 29 | MM: 29  RM: 0 | 24 (83) | 20 (69) | 9 (31) | NR | *C. jejuni + C. coli* :15 (51.7)  *Campylobacter ureolyticus:* 7 (24.1)  *C. fetus: 2* (6.9)  *Campylobacter curvus: 1* (3.6)  *Campylobacter lari: 1* (3.6) | 11 (37.9) | M: 1 (5.6)  Q :6 (33.3) | NR | NR | NR |
| Meda et al, 2024 (44) | 12 | MM: 12  RM: 0 | 8 (67) | 2 (17) | 6 (50) | NR | *C. coli:* 1 (8)  *C. fetus:* 1 (8)  *C. jejuni:* 6 (50)  Other*:* 4 (34) | NR | NR | NR | NR | NR |
| Baek et al, 2024 (45) | 108 | MM: 108  RM: 0 | 98 (91) | 47 (44) | 44) | Soft tissue: 9 (8.3) Pneumonia: 9 (8.3)  Bone/joint infection: 5 (4.6)  Meningoencephalitis: 5 (4.6)  Endovascular: 2 (1.9)  Thrombophlebitis: 1 (0.9) | *C. coli:* 7 (6.5)  *C. fetus:* 21 (19.4)  *C. jejuni:* 54 (50)  *Campylobacter concisus:* 7 (6.5)  *Campylobacter rectus:* 2 (1.9)  NR :17 (15.7) | NR | A 3 (50)  AC 2 (33)  AG 2 (33)  CEF 1 (33)  M 3 (4)  Q 45 (59)  T 13 (28) | A: 3 (50)  AC: 2 (33)  AG: 2 (33) CEF: 1 (33)  M: 3 (4)  Q: 45 (59)  T: 13 (28) | A: 1 (100)  AC: 1 (100)  AG: 1 (100)  CEF: 1 (100)  M: 1 (20)  Q: 4 (67)  T: 2 (40) | Q: 1 (7)  T: 1 (14) |
| Graham, 2024 (35) | 34 | MM: 34  RM: 34 | 12 (36.4) | 22 (66.7) | 12 (36.4) | NR | *C. coli:* 2 (6)  *C. fetus:* 2 (6)  *C. jejuni:* 24 (71) | 11 (36.7) | Q: 14 (45) | NR | Q: 11 (79) | NR |
| Zayet et al, 2023 (25) | 21 | MM: 21  RM: 21 | 13 (62) | 6 (29) | 4 (19) | Mycotic aneurism: 3 (14.3)  Endocarditis: 1 (4.8)  Infected device: 1 (4.8)  Thrombophlebitis: 1 (4.8)  Bone/joint infection 1 (4.8) | *C. fetus*: 21 (100) | 1 (4.8) | NR | NR | NR | Am: 2 (10)  M: 2 (10)  Q: 6 (33) |
| Otsuka et al., 2023 (33) | 39 | MM: 39  RM: 0 | 35 (89.7) | 21 (53.8) | 17 (43.6) | Cellulitis: 2 (5.13)  Mycotic aneurysm: 2 (5.1)  Bone and joint infection: 2 (5.1) | *C. coli:* 4 (10.3)  *C. fetus:* 5 (12.8*)*  *C. jejuni:* 27 (69.2)  *C. lari:* 1 (2.6)  *C. ureolyticus:* 1 (2.6)  Not identified: 1 (2.6) | NR | NR | NR | NR | NR |
| Tinévez et al., 2023 (14) | 57 | MM: 0  RM: 0 | 32 (71.1) | 10 (22.2) | 23 (51.1) | Bone and joint infection: 4 (8.9) | *C. fetus:* 47 (82.5)  *C. jejuni:* 6 (10.5)  Others: 4 (7.5) | NR | NR | NR | AC: 0  Am: 3 (60)  M: 0  Q: 3 (60)  T: 4 (80) | AC: 0  Am: 1 (2.3)  C: 0  M: 1 (2.3)  Q: 11 (25)  T: 5 (13.2) |
| Tinévez et al., 2022 (12) | 592 | MM: 592  RM: 592 | 426 (75.3) | 233 (41.5) | 112 (58.6) | Endovascular: 38 (6.6)  Endocarditis: 12 (2.1)  Bone and joint infection: 24 (4.2)  Peritonitis 9 (1.5)  Meningitis 2 (0.3) | *C. coli:* 40 (6.8)  *C. fetus:* 252 (42.6)  *C. jejuni:* 254 (42.9)  Other: 46 (7.8) | 160 (57.8) | A: 131 (25.4)  AC: 2 (0.6)  AG: 3 (0.6)  C: 0  M: 22 (4)  Q: 249 (45.8)  T: 167 (33.7) | A: 21 (60)  AC: 0  AG: 0  C: 0  M: 9 (23.7)  Q: 22 (57.9)  T: 23 (63.9) | A: 85 (38.5)  AC: 2 (0.8)  AG: 1 (0.4)  C: 0  M: 6 (2.4)  Q: 146 (58.6)  T: 108 (49.5) | A: 17 (7.2)  AC: 0  AG: 2 (0.9)  C: 0  M: 4 (1.7)  Q: 68 (29.4)  T: 33 (15.2) |
| Tau et al., 2022 (5) | 76 | MM: 76  RM: 76 | 64 (84.2) | 50 (65.8) | 22 (28.9) | NR | *C. coli:* 11 (14.5)  *C. jejuni:* 63 (82.9)  Unknown: 2 (2.6) | NR | M: 2 (2.9)  Q: 55 (78.6) | NR | NR | NR |
| Shahunja et al., 2020 (40) | 9 | MM: 9  RM: 0 | NR | NR | NR | NR | NR | 2 (22.2) | A: 4 (50)  AC: 4 (50)  AG: 1 (100)  CEF: 1 (100)  M: 3 (50)  Q: 6 (75) | NR | NR | NR |
| Liu et al., 2019 (38) | 56 | MM: 56  RM: 0 | NR | NR | NR | NR | *C. coli:* 26 (46)  *C. fetus:* 19 (34)  *C. jejuni:* 11 (19.6) | NR | NR | NR | NR | NR |
| O'Hara et al. 2017 (15) | 41 | MM: 41  RM: 0 | 35 (85) | 19 (46) | 13 (32) | NR | *C. coli:* 3 (7.3)  *C. fetus:* 2(4.9)  *C. jejuni:* 20 (48.8)  *Campylobacter hyointestinalis:* 1 (2.4)  *Campylobacter upsaliensis:* 1 (2.4)  *C. ureolyticus:* 1 (2.4)  NR*:* 13 (31.7) | NR | NR | NR | NR | NR |
| Yamamoto et al, 2017 (34) | 14 | MM: 14  RM: 14 | 13 (92.9) | 7 (50) | NR | Cholangitis: 1 (7.14) | *C. fetus:* 2 (14.3) *C. jejuni:* 12 (85.7) | NR | NR | NR | NR | NR |
| Marchand- Senécal et al., 2017 (39) | 3 | MM: 3  RM: 0 | NR | NR | NR | Mycotic aneurysm: 2 (66.6) | *C. fetus:* 3 (100) | NR | NR | NR | NR | NR |
| Cypierre et al., 20114 (26) | 20 | MM: 29  RM: 0 | 14 (70) | 0 | 5 (25) | Soft tissue infection: 3 (15)  Septic arthritis: 3 (15)  Pneumonia: 4 (20) | *C. fetus:* 100% | NR | A: 4 (20)  AC: 0  AG: 0  M: 0  Q: 6 (30) | NR | NR | NR |
| Mori et al., 2014 (32) | 7 | MM: 7  RM: 7 | 7 (100) | 2 (28.6) | NR | NR | *C. jejuni:* 7 (100) | NR | NR | NR | NR | NR |
| Ben-Shimol et al., 2013 (10) | 60 | MM: 60  RM: 0 | NR | NR | NR | NR | *C. coli*: 19 (31.7)  *C. jejuni*: 27 (45)  *C. lari:* 1 (1.7) | 10 (16.7) | NR | AG 4 (40) | Q 5 (38.5) | NR |
| Liao et al., 2012 (9) | 24 | MM: 24  RM: 0 | 14 (58) | 8 (33) | 7 (29) | NR | *C. coli:* 15 (65.2)  *C. fetus:* 6 (25)  *C. jejuni:* 3 (12.5) | 1 (4.2) | C: 3 (12.5)  CEF 24 (100)  Q:15 (62.5) | NR | NR | NR |
| Feodoroff et al., 2011 (16) | 76 | MM: 76  RM: 0 | 64 (84) | 60 (79) | NR | NR | *C. coli:* 3 (4)  *C. jejuni:* 73 (96) | NR | NR | NR | NR | NR |
| Fica et al., 2011 (42) | 6 | MM: 6  RM: 0 | 3 (50) | 5 (83.3) | 1 (16.7) | NR | *C. fetus:* 5 (71.4)  *C. jejuni:* 2 (28.6) | NR | NR | NR | NR | NR |
| Nielsen et al., 2010 (3) | 46 | MM: 46  RM: 46 | NR | 27 (59%) | NR | Soft tissue: 1 (50) Endocarditis: 1 (50) | *C. coli*: 5 (10.8)  *C. fetus*: 3 (6.5)  *C. jejuni*: 37 (80.4)  *C. lari:* 1 (2.2) | NR | NR | NR | NR | NR |
| Fernández-Cruz et al., 2010 (29) | 68 | MM: 68  RM: 68 | 55 (80.9) | 23 (33.8) | 24 (35.3) | Cellulitis: 4 (5.9)  Pneumonia: 8 (11.8) | *C. coli:* 8 (11.8)  *C. fetus:* 13 (19.1)  *C. jejuni:* 45 (66.2)  NR: 2 (3) | 8 (14.8) | AC: 2 (5.7)  AG: 2 (5.7)  C: 0  CEF: 7 (22.6)  CTM: 13 (36.1)  M: 4 (13)  Q: 19 (50) | AG: 1 (33)  CEF: 1 (50)  CTM: 2 (50) | AC: 2 (8.3)  AG: 1 (4.2)  CEF: 5 (20.8)  CTM: 6 (25)  M: 3 (11.5)  Q: 19 (70.4) | CEF: 1 (20)  CTM: 5 (62.5)  M: 1 (12.5) |
| Cochennec et al.,2008 (27) | 5 | MM: 0  RM: 0 | 5 (100) | 1 (20) | 5 (100) | Mycotic aneurysm: 5 (100) | *C. fetus:* 5 (100) | NR | NR | NR | NR | M: 1 (20)  Q: 1 (20)  T: 2 (50) |
| Gazaigne et al., 2008 (7) | 21 | MM: 21  RM: 21 | 12 (57) | 1 (4.8) | 3 (14.3) | Endocarditis: 4.8%  Cellulitis: 19%  Meningitis: 1 (4.8)  Mycotic aneurysm: 5 (23.8)  Bone/joint infection: 1 (4.8)  Peritonitis: 1 (4.8) | *C. fetus:* 21 (100) | NR | NR | NR | NR | NR |
| Pacanowski et al., 2008 (13) | 178 | MM: 178  RM: 0 | 74 (42) | 58 (33) | NR | Soft tissue: 28 (16)  Endovascular: 13 (7)  Osteoarticular: 5 (3)  Infected device: 7 (4) | *C. coli*: 16 (9)  C*. fetus*: 94 (53)  *C. jejuni*: 54 (30.3)  *C. lari:* 2 (1.1) | 13 (21) | NR | NR | NR | A: 2 (2)  AC: 0  M: 3 (3)  Q: 21 (30) |
| Woo et al.,2002 (41) | 8 | MM: 8  RM: 0 | 6 (75) | 2 (25) | 3 (37.5) | Soft tissue: 2 (25) | *C. fetus*: 8 (100) | NR | NR | NR | NR | A: 2 (25)  AG: 0 (0)  C: 0 (0)  M: 4 (50)  Q: 2 (25) |
| Tee et al., 1998 (43) | 21 | MM: 21  RM: 0 | 19 (90.5) | 19 (90.5) | NR | Pneumonia: 7 (33.3)  Soft tissue: 3 (14.3) | *C. jejuni*: 21 (100) | NR | NR | NR | Q: 3 (14.3) | NR |
| Pigrau et al., 1997 (30) | 58 | MM: 58  RM: 58 | 53 (91) | 18 (33) | NE | Soft tissue: 5 (9)  Pneumonia: 2 (4)  Meningoencephalitis: 1 (2)  Pancreatitis: 1 (2) | *C. coli:* 1 (2)  *C. fetus:* 4 (7)  *C. jejuni:* 47 (81)  Others: 6 (10) | 15 (40) | Am: 5 (20)  AC: 1 (4)  CTM: 18 (79)  M: 4 (7)  Q: 13 (54) | NR | AC :1 (4.6)  CEF: 34 (100)  CTM: 13 (72.2)  M: 3 (6.4)  Q: 12 (66.7) | CTM: 2 (100)  Q: 1 (50 |
| Font et al.1997 (28) | 30 | MM: 30  RM: 30 | 30 (100) | 14 (46) | NR | Soft tissue: 1 (3.3)  Mycotic aneurysm: 1 (3.3) | *C. fetus*: 4 (13.3)  *C. jejuni*: 26 (86.7) | 10 (33.3) | NR | NR | Q: 16 (54) | NR |
| Pigrau et al., 1996 (31) | 7 | MM: 0  RM: 0 | 7 (100) | 4 (29) | NR | Soft tissue: 1 (14)  Pneumonia:1 (14) | *C. jejuni:* 5 (71)  NR: 2 (29) | 2 (29) | A: 5 (23)  AG: 0  CTM: 16 (76)  M: 4 (7)  Q: 10 (52) | NR | NR | NR |
| Schonheyder et al., 1995 (37) | 15 | MM: 15  RM: 15 | 15 (100) | 8 (53.3) | NR | Soft tissue: 1 (6.67)  Cholangitis: 1 (6.67) | *C. coli: 6* (40)  *C. fetus:* 2 (13.3)  *C. jejuni:* 6 (40) | NR | NR | NR | NR | NR |
| Skirrow et al., 1993 (36) | 394 | MM: 394  RM: 0 | NR | 168 (67.2) | NR | NR | *C. coli:* 23 (5.8)  *C. fetus:*  22 (5.6)  *C. jejuni*: 91 (23.1)  NR: 137 (34.7) | NR | NR | NR | NR | NR |

MM: Mortality meta-analysis; RM: relapse meta-analysis; R: resistance; A: amoxicillin; Am: ampicillin; AC: amoxicillin–clavulanic acid; ß-lact: β-lactam antibiotic; C: carbapenems; CLI: clindamycin; M: macrolides; Q: quinolones; CTM: cotrimoxazole; AG: aminoglycosides; CEF: cephalosporins; Dox: doxycycline; P: pristinamycin; T: tetracycline; TC: tigecycline; Met: metronidazole; V: vancomycin; NR: not reported.

**Supplemental Table S6.** Treatment and prognosis of *Campylobacter* spp. BSI included in 32 original research articles.

| **Study** | **n** | **N patients included in mortality or relapse meta-analyses** | **Monotherapy/**  **Combined treatment**  **n (%)** | **Monotherapy treatment**  **n (%)** | **Combined therapy**  **n (%)** | **Duration of treatment (days)** | **Hospital admission**  **n (%)** | **Relapse**  **n (%)** | **Global mortality**  **n (%)** | **Mortality according to species**  **n (%)** |
| --- | --- | --- | --- | --- | --- | --- | --- | --- | --- | --- |
| Sunnerhagen et al, 2024 (17) | 29 | MM: 29  RM: 0 | NR | NR | NR | 13 (9–17) | 28 (97) | NR | 4 (14) | NR |
| Meda et al, 2024 (44) | 12 | MM: 12  RM: 0 | NR | NR | NR | NR | NR | NR | 1 (8) | NR |
| Baek et al, 2024 (45) | 108 | MM: 108  RM: 0 | NR | NR | NR | NR | NR | NR | 14 (13.0) | *C. coli:* 0  *C. fetus:* 3 (14.3)  *C. jejuni*: 3 (5.6)  NR: 6 (35.3) |
| Graham et al, 2024 (35) | 34 | MM: 34  RM: 34 | MT: 28 (85.3)  CT 6 (17.1) | NR | NR | 13 (10-14) | 32 (94.1) | 1 (3) | 1 (3) | NR |
| Zayet et al, 2023 (25) | 21 | MM: 21  RM: 21 | MT: 13 (61.9)  CT: 8 (38.1) | NR | AC+AG: 3 (38)  AC+M: 1 (12)  AC+Q: 2 (25)  AC+Dox: 1 (12)  C+AG:1 (12) | 9 (SD 8) | 21 (100) | 2 (9.5) | 7 (33) | NR |
| Otsuka et al., 2023 (33) | 39 | MM: 39  RM: 0 | Intravenous treatment: 26 (66.7) | NR | NR | 9 (4-15) | 35 (89.7) | NR | 0 | NR |
| Tinévez et al., 2023 (14) | 57 | MM: 0  RM: 0 | Vascular or prosthetic valves infection:  - MT 9/24 (37.5)  - CT 15/24 (62.5)  Endocarditis group:  CT 100% | NR | Valvular infection:  -ß-lact+AG 5 (33.3)  -ß-lact+ M 1 (6.7)  -ß-lact+Q 9 (60)  Endocarditis:  -AG+ß-lact 11 (91.7)  -AG+Q: 1 (7.7) | 42 (IQR 20–49 days) | NR | 4 (8.2) | 12 (24.5) | NR |
| Tinévez et al., 2022 (12) | 592 | MM: 592  RM: 592 | NR | NR | NR | NR | NR | 29 (4.9) | 69 (11.7) | *C. coli:* 1 (2.5)  *C. fetus:* 29 (11.5)  *C. jejuni:* 28 (11) |
| Tau et al., 2022 (5) | 76 | MM: 76  RM: 76 | NR | NR | NR | NR | NR | 0 | 11 (14.4) | NR |
| Shahunja et al., 2020 (40) | 9 | MM: 9  RM: 0 | NR | NR | NR | NR | NR | NR | 0 | NR |
| Liu et al., 2019 (38) | 56 | MM: 56  RM: 0 | NR | NR | NR | NR | NR | NR | 3 (5.4) | *C. coli:* 2 (7.7)  *C. fetus:* 1 (5.3)  *C. jejuni:* 0 |
| O'Hara et al, 2017 (15) | 41 | MM: 41  RM: 0 | MT: 5 (17.2)  CT: 16 (55.7) | Q: 5 (100) | CEF+Met: 4 (13.8)  NR: 12 (41.3) | NR | NR | NR | 2 (4.9) | NR |
| Yamamoto et al, 2017 (34) | 14 | MM: 14  RM: 14 | NR | NR | NR | 10 ± 8 | NR | 0 | 0 | NR |
| Marchand- Senécal et al., 2017 (39) | 3 | MM: 3  RM: 0 | NR | NR | NR | NR | NR | NR | 0 | *C. fetus:* 0 |
| Cypierre et al., 2014 (26) | 20 | MM: 29  RM: 0 | MT: 7 (35)  CT: 12 (60) | A/AC: 3 (15)  Q: 2 (10)  P: 2 (10) | A/AC+AG: 3 (15)  A/AC+Q: 3 (15)  A/AC+M: 2 (10)  A/AC+AG+Q: 2 (10)  AG+Q: 1 (5)  AG+Q+M: 1 (5) | 19 (5-51) | NR | NR | 1 (5) | NR |
| Mori et al., 2014 (32) | 7 | MM: 7  RM: 7 | MT: 3 (43)  CT: 2 (28.6) | C: 1 (33.3)  M: 1 (33.3)  Q: 1 (33.3) | C+Q: 1 (50)  CEF+Q: 1 (50) | NR | NR | 0 | 0 | NR |
| Ben-Shimol et al., 2013 (10) | 60 | MM: 60  RM: 0 | NR | NR | NR | NR | NR | NR | 0 | NR |
| Liao et al., 2012 (9) | 24 | MM: 24  RM: 0 | MT: 24 (100)  CT: 0 (0) | AC: 3 (13)  CEF: 13 (54)  Q: 5 (21) | NR | NR | NR | NR | 3 (13) | *C. coli:* 2 (13)  *C. fetus*: 0  *C. jejuni:* 0 |
| Feodoroff et al., 2011 (16) | 76 | MM: 76  RM: 0 | NR | NR | NR | NR | NR | NR | 6 (8) | NR |
| Fica et al., 2011 (42) | 6 | MM: 6  RM: 0 | MT: 1 (16.6)  CT: 4 (66.6) | CEF: 1 (100) | CEF+ M: 1 (25)  CEF + Met: 1 (25)  M+AG+CEF: 1 (25)  Q+Met: 1 (25) | NR | NR | NR | 2 (33.3) | *C. fetus:* 2 (100)  *C. jejuni:* 0 |
| Nielsen et al., 2010 (3) | 46 | MM: 46  RM: 46 | NR | NR | NR | NR | NR | 0 | 3 (6) | NR |
| Fernández-Cruz et al., 2010 (29) | 68 | MM: 68  RM: 68 | NR | NR | NR | NR | NR | 7 (10.3) | 10 (14.7) | NR |
| Cochennec et al.,2008 (27) | 5 | MM: 0  RM: 0 | NR | NR | NR | NR | NR | NR | 3 (60) | NR |
| Gazaigne et al., 2008 (7) | 21 | MM: 21  RM: 21 | MT: 10 (47.6)  CT: 11 (52.4) | AC: 5 (50)  A: 1 (10)  C: 2 (20)  Q: 1 (10)  P: 1 (10) | AC+M: 3 (27.3)  AC+Q: 3 (27.3)  AC+AG: 1 (9.1)  C+AG: 1 (9.1)  C+A: 1 (9.1)  C+AC: 1 (9.1)  C+Q: 1 (9.1) | 28 (9 days-3.5 months) | NR | 2 (9.5) | 3 (14.3) | *C. fetus*: 3 (14.3) |
| Pacanowski et al., 2008 (13) | 178 | MM: 178  RM: 0 | NR | NR | NR | NR | NR | NR | 27 (15) | *C. fetus:* 13 (14)  No *C. fetus:* 13 (17) |
| Woo et al.,2002 (41) | 8 | MM: 8  RM: 0 | MT: 5 (62.5)  CT: 3 (37.5) | AC: 2 (40)  CEF: 2 (40)  TIC: 1 (20) | Penicillin+AG: 3 (100) | NR | NR | NR | 2 (25) | *C. fetus*: 2 (25) |
| Tee et al., 1998 (43) | 21 | MM: 21  RM: 0 | MT 8 (38.1)  CT 12 (51.1) | M: 2 (25)  Q: 6 (75) | AG+CEF: 1 (8.3)  AG+CLI: 1 (8.3)  AG+M+A: 1 (8.3)  CEF+M: 1 (8.3)  CEF+AG+Q: 2 (16.7)  Q+M: 2 (16.7)  Q+M+AG: 1 (8.3)  TC+M+AG: 2 (16.7) | NR | NR | NR | 3 (14.3) | *C. jejuni:* 4 (19.5) |
| Pigrau et al., 1997 (30) | 58 | MM: 58  RM: 58 | NR | NR | NR | 1-3 weeks | 16 (30) | 1 (1.7) | 6 (10.34) | *C. coli*: 0  *C. fetus*: 0  *C. jejuni:* 6 (13) |
| Font et al., 1997 (28) | 30 | MM: 30  RM: 30 | NR | NR | NR | NR | NR | 0 | 3 (10) | NR |
| Pigrau et al., 1996 (31) | 7 | MM: 0  RM: 0 | NR | NR | NR | NR | NR | 1 (14) | 0 | NR |
| Schonheyder al., 1995 (37) | 15 | MM: 15  RM: 15 | MT: 3 (20)  CT: 6 (40) | CEF: 1 (33.3)  Penicillin: 2 (66.7) | Penicillin or Am+AG: 100% | NR | 8 (53.3) | 2 (13.3) | 1 (6.7) | NR |
| Skirrow et al., 1993 (36) | 394 | MM: 394  RM: 0 | NR | NR | NR | NR | NR | NR | 10 (2.5) | NR |

MM: Mortality meta-analysis; RM: relapse meta-analysis; MT: monotherapy; CT: combination therapy; A: amoxicillin; Am: ampicillin; AC: amoxicillin–clavulanic acid; ß-lact: β-lactam antibiotic; C: carbapenems; CLI: clindamycin; M: macrolides; Q: quinolones; CTM: cotrimoxazole; AG: aminoglycosides; CEF: cephalosporins; Dox: doxycycline; P: pristinamycin; T: tetracycline; TIC: ticarcillin; TC: tigecycline; Met: metronidazole; V: vancomycin; ICU: intensive care unit; NR: not reported.

**Supplemental Table S7.** Meta-regression analyses based on the proportion of participants included in the primary research studies with different *Campylobacter* spp.

| **Proportion of participants**  **included in the studies**  **with different**  ***Campylobacter* species** | **Number**  **of studies** | **Estimate (95% CI)** | **p-value** | **Residual**  **heterogeneity** |
| --- | --- | --- | --- | --- |
| **Association with MORTALITY** |  |  |  |  |
| ***C. jejuni*** | 27 | -0.0009 (-0.0025, 0.0008) | 0.2997 | 72.3% |
| - Age- and sex-adjusted | 24 | -0.0014 (-0.0029, 0.0001) | 0.0701 | 40.6% |
| ***C. fetus*** | 26 | 0.0020 (0.0007, 0.0034) | 0.0032 | 47.8% |
| - Age- and sex-adjusted | 24 | 0.0023 (0.0008, 0.0037) | 0.0019 | 0.1% |
| ***C. coli*** | 27 | -0.0015 (-0.0048, 0.0017) | 0.3548 | 71.3% |
| - Age- and sex-adjusted | 24 | -0.0002 (-0.0033, 0.0029) | 0.8898 | 44.3% |
| **Association with RELAPSE** |  |  |  |  |
| ***C. jejuni*** | 12 | -0.0028 (-0.0048, -0.0009) | 0.0038 | 29.9% |
| - Age- and sex-adjusted | 12 | -0.0037 (-0.0056, -0.0018) | 0.0002 | 0.0% |
| ***C. fetus*** | 11 | 0.0018 (-0.0001, 0.0036) | 0.0681 | 30.4% |
| - Age- and sex-adjusted | 11 | 0.0022 (-0.0006, 0.0049) | 0.1179 | 44.8% |
| ***C. coli*** | 12 | 0.0028 (-0.0047, 0.0103) | 0.4632 | 63.4% |
| - Age- and sex-adjusted | 12 | 0.0042 (-0.0046, 0.0131) | 0.3472 | 67.7% |

**Supplemental Table S8.** Baseline characteristics and prognosis of *Campylobacter* spp. bloodstream infections published as individual cases.

|  | **TOTAL***  **n= 212** | ***C. coli***  **n=22** | ***C. jejuni***  **n=74** | ***C. fetus***  **n=89** | **P value** |
| --- | --- | --- | --- | --- | --- |
| **BASELINE CHARACTERISTICS** | | | | | |
| **Age in years – median (IQR)** | 54  (33-70) | 40.5  (32-57.75) | 36.5  (20.75-57.25) | 63.5  (46.25 -75) | **<0.001** |
| **Sex (male) *–* n (%)** | 134 (63.2) | 18 (81.8) | 43 (58.1) | 53 (59.6) | **0.35** |
| **Comorbidity – n (%)** | 147 (69.3) | 12 (54.5) | 56 (75.7) | 63 (70.8) | 0.15 |
| **Excessive alcohol consumption – n (%)** | 10 (4.7) | 0 (0) | 4 (5.4) | 6 (6.7) | 0.39 |
| **Diabetes– n (%)** | 25 (11.8) | 0 (0) | 5 (6.8) | 18 (20.2) | **0.013** |
| **Renal insufﬁciency – n (%)** | 18 (8.5) | 1 (4.5) | 6 (8.1) | 8 (9) | 0.75 |
| **Liver disease – n (%)** | 23 (10.8) | 3 (13.6) | 11 (14.9) | 7 (7.9) | 0.37 |
| **Connective tissue disease – n (%)** | 2 (0.9) | 0 (0) | 1 (1.4) | 1 (1.1) | 0.90 |
| **Active solid neoplasm – n (%)** | 10 (4.7) | 2 (9.1) | 5 (6.8) | 2 (2.2) | 0.43 |
| **Active haematological malignancy – n (%)** | 25 (11.8) | 1 (4.5) | 12 (16.2) | 9 (10.1) | 0.40 |
| **Neutropenia <500.106/l – n (%)** | 2 (0.9) | 1 (4.5) | 1 (1.4) | 0 (0) | **0.25** |
| **Immunotherapy with biologics in the last 6 months – n (%)** | 5 (2.4) | 1 (4.5) | 4 (5.4) | 0 (0) | 0.11 |
| **Solid organ transplantation – n (%)** | 6 (2.8) | 0 (0) | 4 (5.4) | 2 (2.2) | 0.38 |
| **Haematopoietic stem cell transplantation – n (%)** | 5 (2.4) | 1 (4.5) | 3 (4.1) | 0 (0) | 0.27 |
| **CAR-T** | 0 (0) | 0 (0) | 0 (0) | 0 (0) | - |
| **Steroids – n (%)** | 23 (10.8) | 1 (4.5) | 9 (12.2) | 11 (12.4) | 0.54 |
| **Immunosuppressive drugs– n (%)** | 26 (12.3) | 1 (4.5) | 15 (20.3) | 9 (10.1) | 0.07 |
| **HIV- n (%)** | 14 (6.6) | 2 (9.1) | 8 (8.1) | 4 (4.5) | 0.78 |
| **Primary or secondary immunodeﬁciency**  **- n (%)** | 97 (45.8) | 13 (59.1) | 48 (64.9) | 29 (32.6) | **<0.001** |
| **Primary immunodeﬁciency *–* n (%)**  **- Primary immunodeﬁciency. types:**   - **Agammaglobulinemia** - **SCID** - **Good syndrome** - **Not specified** | 25 (11.8)  15 (7.1)  3 (1.4)  4 (1.9)  2 (0.9) | 7 (31.8)  5 (22.7)  1 (4.5)  0 (0)  1 (4.5) | 13 (17.6)  9 (12.2)  2 (2.7)  1 (1.4)  1 (1.4) | 4 (4.5)  0 (0)  0 (0)  3 (3.4)  1 (1.1) | **<0.001** |
| **Secondary humoral immunodeﬁciency – n (%)** | 34 (16.0) | 2 (9.1) | 17 (23) | 12 (13.5) | **0.03** |
| **Primary or secondary humoral immunodeﬁciency - n (%)** | 60 (28.3) | 9 (40.9) | 31 (41.9) | 16 (18) | **<0.001** |
| **CLINICAL FINDINGS** | | | | | |
| **Fever – n (%)** | 158 (74.5) | 17 (77.3) | 57 (77) | 67 (75.3) | 0.86 |
| **Cough – n (%)** | 17 (8.0) | 0 (0) | 7 (9.5) | 6 (6.7) | 0.34 |
| **Diarrhea – n (%)** | 61 (28.8) | 7 (31.8) | 29 (39.2) | 20 (22.5) | **0.04** |
| **Vomiting – n (%)** | 16 (7.5) | 0 | 8 (10.8) | 6 (6.7) | 0.39 |
| **Abdominal pain- n (%)** | 33 (15.6) | 3 (13.6) | 18 (24.3) | 10 (11.2) | 0.10 |
| **Weight loss – n (%)** | 14 (6.6) | 0 (0) | 4 (5.4) | 8 (9) | 0.44 |
| **Arthralgia – n (%)** | 14 (6.6) | 1 (4.5) | 3 (4.1) | 6 (6.7) | 0.43 |
| **Skin symptoms - n (%)** | 28 (13.7) | 4 (18.2) | 8 (10.8) | 12 (13.5) | 0.77 |
| **Neurological symptoms- n (%)** | 14 (6.6) | 1 (4.5) | 1 (1.4) | 8 (9) | **0.03** |
| **Sepsis – n (%)** | 12 (5.7) | 0 (0) | 8 (10.8) | 4 (4.5) | 0.09 |
| **Immune-mediated symptoms - n (%)** | 7 (3.3) | 1 (4.5) | 5 (6.8) | 1 (1.1) | 0.19 |
| **Lung filtrate - n (%)** | 14 (6.6) | 0 (0) | 1 (5.4) | 5 (5.6) | **0.012** |
| **Extra-intestinal disease - n (%)** | 95 (44.8) | 8 (36.4) | 20 (27) | 50 (56.2) | **<0.001** |
| - **Cellulitis – n (%)** | 23 (10.8) | 2 (9.1) | 9 (12.2) | 8 (9) | 0.92 |
| - **Endocarditis – n (%)** | 12 (5.7) | 1 (4.5) | 0(0) | 11 (12.4) | **0.005** |
| - **Endovascular (non-endocarditis)- n (%)** | 10 (4.7) | 0 (0) | 1 (1.4) | 9 (10.1) | **0.022** |
| - **Osteomyelitis n (%)** | 7 (3.3) | 0 (0) | 1 (1.4) | 3 (3.4) | **0.032** |
| - **Septic arthritis- n (%)** | 7 (3.3) | 2 (9.1) | 1 (1.4) | 2 (2.2) | 0.13 |
| - **Meningitis- n (%)** | 11 (5.2) | 1 (4.5) | 0(0 | 9 (10.1) | **0.041** |
| - **Pneumonia- n (%)** | 11 (5.2) | 0 (0) | 3 (4.1) | 4 (4.5) | **0.032** |
| **Isolation in feces (n=73) - n (%)** | 40/73 (54.8) | 7/10 (70) | 22/34 (64.7) | 10/25 (40) | **0.044** |
| **ANTIMICROBIAL THERAPY** | | | | | |
| **Monotherapy – n (%)** | 102 (48.1) | 11 (50) | 36 (48.6) | 43 (48.3) | 0.82 |
| - **Length. days – median (IQR)** | 21 (14-40) | 16 (10-28) | 14 (10-28) | 28 (16.5-42) | 0.25 |
| **Combination therapy – n (%)** | 88 (41.5) | 9 (40.9) | 30 (40.5) | 38 (42.7) | 0.16 |
| - **Length. days – median (IQR)** | 26.5  (13.5-42) | 35 (21-57) | 14 (7-21) | 35  (19.5-42) | **0.002** |
| **OUTCOMES** | | | | | |
| **Microbiological cure. day 30 -n (%)** | 47 (88.7) | 4 (100) | 13 (81.3) | 27 (96.4) | 0.07 |
| **30-day mortality -n (%)** | 17 (8.8) | 0 (0) | 7 (11.1) | 6 (7.1) | 0.17 |
| **Mortality- n (%)** | 22 (11.3) | 0 (0) | 10 (15.6) | 8 (9.4) | 0.17 |
| **Relapse- n (%)** | 23 (12.5) | 7 (35) | 10 (17.2) | 4 (4.9) | **0.001** |
| - **Number of relapses – median (IQR)** | 2 (1-3) | 3 (3-3) | 1.5 (1-3) | 1 (1-2) | **0.12** |

*Some *Campylobacter* spp. BSI included in the table were causes by species other than *C. coli*, *C. jejuni* or *C. fetus*, or the *Campylobacter* species was not reported in the included studies.

PID: Primary immunodeﬁciency; SCID*:* severe combined immunodeficiency; CAR-T: Chimeric antigen receptor T-cell therapy

**Supplemental Table S9**. Microbiological characteristics of *Campylobacter* spp. in individual published clinical cases.

|  | **TOTAL***  **n=212** | ***C. coli***  **n=22** | ***C. jejuni***  **n= 74** | ***C. fetus***  **n=89** | **P value** |
| --- | --- | --- | --- | --- | --- |
| **Amoxicillin resistance – n (%)** | 6/47 (12.8) | 1/5 (20) | 5/12 (41.7) | 0/27 (0) | **0.003** |
| **Amoxicillin-clavulanic acid resistance - n (%)** | 2/20 (10) | 1/2 (50) | 1/5 (20) | 0/12 (0) | 0.14 |
| **Cephalosporin resistance-n (%)** | 13/24 (54.2) | 1/3 (33.3) | 6/9 (66.7) | 3/8 (37.5) | 0.44 |
| **Macrolide resistance - n (%)** | 16/75 (21.3) | 5/13 (38.5) | 6/28 (21.4) | 3/29 (10.3) | 0.10 |
| **Quinolone resistance – n (%)** | 46/85 (54.1) | 6/11 (54.6) | 22/31 (70.9) | 12/36 (33.3) | **0.002** |
| **Tetracycline resistance – n (%)** | 10/33 (30.3) | 1/2 (50) | 5/10 (50) | 4/20 (20) | 0.30 |
| **Aminoglycoside resistance - n (%)** | 4/49 (8.2) | 1/4 (25) | 3/17 (17.7) | 0/26 (0) | 0.11 |
| **Fosfomycin resistance - n (%)** | 2/8 (25) | 0/3 (0) | 1/3 (33.3) | 0/1 (0) | 0.22 |
| **Cotrimoxazole resistance - n (%)** | 4/11 (36.4) | 0/2 (0) | 1/4 (25) | 2/4 (50) | 0.33 |
| **Carbapenem resistance- n (%)** | 2/40 (5) | 2/8 (25) | 0/12 (0) | 0/18 (0) | **0.04** |

*Some *Campylobacter* spp. BSI included in the table were causes by species other than *C. coli*, *C. jejuni* or *C. fetus*, or the *Campylobacter* species was not reported in the included studies.

**Supplemental Table S10.** Baseline characteristics and prognosis of *Campylobacter spp*. bloodstream infection published as individual cases according to immunological status.

|  | **TOTAL***  **n= 212** | **PID**  **n=25** | **Non-PID n=159** | **p-value**** |
| --- | --- | --- | --- | --- |
| **BASELINE CHARACTERISTICS** | | | | |
| **Age in years – median (IQR)** | 54  (33-70) | 26  (18.5-44.7) | 57  (38-71) | **<0.001** |
| **Sex (male) *–* n (%)** | 134 (63.2) | 21 (84) | 100 (62.9) | 0.08 |
| **Comorbidity- n (%)** | 147 (69.3) | 8 (32) | 123 (77.4) | **<0.001** |
| **Excessive alcohol consumption – n (%)** | 10 (4.7) | 0 (0) | 6 (3.8) | 0.32 |
| **Diabetes – n (%)** | 25 (11.8) | 0 (0) | 23 (14.5) | **0.042** |
| **Renal insufficiency – n (%)** | 18 (8.5) | 0 (0) | 17 (10.7) | 0.086 |
| **Liver disease – n (%)** | 23 (10.8) | 2 (8) | 17 (10.7) | 0.68 |
| **Connective tissue disease – n (%)** | 2 (0.9) | 0 (0) | 2 (1.3) | 0.57 |
| **Active solid neoplasm – n (%)** | 10 (4.7) | 0 (0) | 8 (5) | 0.25 |
| **Active hematologic malignancy – n (%)** | 25 (11.8) | 0 (0) | 24 (15.1) | **0.037** |
| **Neutropenia <500.106/l – n (%)** | 2 (0.9) | 0 (0) | 2 (1.3) | 0.57 |
| **Biologic immunotherapy in the past 6 months – n (%)** | 5 (2.4) | 0 (0) | 5 (3.1) | 0.37 |
| **Solid organ transplant – n (%)** | 6 (2.8) | 0 (0) | 6 (3.8) | 0.32 |
| **Hematopoietic stem cell transplantation – n (%)** | 5 (2.4) | 0 (0) | 4 (2.5) | 0.42 |
| **Steroids – n (%)** | 23 (10.8) | 1 (4) | 22 (13.8) | 0.17 |
| **CLINICAL FINDINGS** | | | | |
| **Fever – n (%)** | 158 (74.5) | 17 (68) | 123 (77.4) | 0.31 |
| **Cough – n (%)** | 17 (8) | 5 (20) | 11 (6.9) | **0.031** |
| **Diarrhea – n (%)** | 61 (28.8) | 5 (20) | 47 (26.9) | 0.32 |
| **Vomiting – n (%)** | 16 (7.5) | 2 (8) | 12 (7.5) | 0.94 |
| **Abdominal pain - n (%)** | 33 (15.6) | 2 (8) | 24 (15.1) | 0.34 |
| **Weight loss – n (%)** | 14 (6.6) | 3 (12) | 10 (6.3) | 0.30 |
| **Arthralgia – n (%)** | 14 (6.6) | 1 (4) | 10 (6.3) | 0.65 |
| **Skin symptoms - n (%)** | 28 (13.7) | 9 (36) | 19 (11.9) | **0.008** |
| **Neurological symptoms- n (%)** | 14 (6.6) | 0 (0) | 14 (8.8) | 0.12 |
| **Sepsis – n (%)** | 12 (5.7) | 0 (0) | 11 (6.9) | 0.18 |
| **Immune-mediated symptoms - n (%)** | 7 (3.3) | 0 (0) | 5 (3.1) | 0.37 |
| **Lung filtrate - n (%)** | 14 (6.6) | 3 (12) | 11 (6.9) | 0.37 |
| **Extra-intestinal disease - n (%)** | 95 (44.8) | 15 (60) | 72 (45.3) | 0.17 |
| - **Cellulitis – n (%)** | 23 (10.8) | 9 (36) | 12 (7.5) | **<0.001** |
| - **Endocarditis – n (%)** | 12 (5.7) | 1 (4) | 10 (6.3) | 0.65 |
| - **Endovascular (Non endocarditis)- n (%)** | 10 (4.7) | 0 (0) | 9 (5.7) | 0.22 |
| - **Osteomyelitis n (%)** | 7 (3.3) | 1 (4) | 6 (3.8) | 0.96 |
| - **Arthritis- n (%)** | 7 (3.3) | 2 (8) | 3 (1.9) | 0.08 |
| - **Meningitis- n (%)** | 11 (5.2) | 0 (0) | 11 (6.9) | 0.18 |
| - **Pneumonia- n (%)** | 11 (5.2) | 1 (4) | 10 (6.3) | 0.65 |
| **ANTIMICROBIAL THERAPY** | | | | |
| **Monotherapy – n (%)** | 102 (48.1) | 10 (40) | 80 (50.3) | 0.55 |
| **- Length, days – median (IQR)** | 21 (14-40) | 28 (15.5-42) | 21 (14-41) | 0.77 |
| **Combination therapy – n (%)** | 88 (41.5) | 11 (44) | 66 (41.5) | 0.41 |
| **- Length, days – median (IQR)** | 26.5  (13.5-42) | 28  (21-52.5) | 26.5  (11.7-42) | 0.29 |
| **OUTCOMES** | | | | |
| **Microbiological cure, day 30 -n/N (%)** | 47/53 (88.7) | 4/5 (80) | 41/46 (89.1) | 0.55 |
| **30-day mortality -n (%)** | 17 (8.8) | 0 (0) | 16 (10.7) | 0.10 |
| **Global mortality- n (%)** | 22 (11.3) | 12 | 19 (12.7) | 0.54 |
| **Relapses- n (%)** | 23 (12.5) | 15 (65.2) | 8 (5.6) | **<0.001** |
| **- Number of relapses – median (IQR)** | 2 (1-3) | 2.50 (1-3) | 1 (1-2.75) | 0.39 |

*Some *Campylobacter* spp. BSI included in the table were causes by species other than *C. coli*, *C. jejuni* or *C. fetus*, or the *Campylobacter* species was not reported in the included studies.

**p-value compares PID vs non-PID patients

PID: Primary immunodeﬁciency

**Supplemental Table S11.** Microbiological characteristics of *Campylobacter* spp. in individual published clinical cases according to immunological status**.**

|  | **Total**  **n=212*** | **PID**  **n=25** | **No PID n=159** | **p-value**** |
| --- | --- | --- | --- | --- |
| **Isolation in feces (n=73) - n (%)** | 40/73 (54.8) | 10/14 (71.4) | 22/50 (44) | 0.07 |
| **Species- n (%)** |  |  |  |  |
| *-C. coli* | 22 (10.4) | 7 (28) | 12 (7.5) | **0.003** |
| *-C. jejuni* | 74 (34.9) | 13 (52) | 46 (28.9) | **0.033** |
| *-C. fetus* | 89 (42) | 4 (16) | 79 (49.7) | **0.001** |
| -Other species | 22 (10.4) | 1 (4) | 17 (11) | 0.28 |
| **Amoxicillin resistance – n (%)** | 6/47 (12.8) | 1/3 (33.3) | 5/38 (13.2) | 0.34 |
| **Amoxicillin-clavulanic acid resistance – n (%)** | 2/20 (10) | 1/4 (25) | 1/14 (7.1) | 0.32 |
| **Cephalosporin resistance-n (%)** | 13/24 (52.2) | 2/3 (66.7) | 9/19 (47.4) | 0.53 |
| **Macrolide resistance - n (%)** | 16/75 (21.3) | 8/13 (61.5) | 7/58 (12.1) | **<0.001** |
| **Quinolone resistance – n (%)** | 46/85 (54.1) | 10/13 (76.9) | 35/67 (52.2) | 0.10 |
| **Tetracycline resistance – n (%)** | 10/33 (30.3) | 1/1(100) | 8/29 (27.6) | 0.12 |
| **Aminoglycoside resistance - n (%)** | 4/49 (8.2) | 0/3(0) | 4/42 (9.5) | 0.58 |
| **Fosfomycin resistance – n (%)** | 2/8 (25) | 0/2(0) | 2/6 (33.3) | 0.35 |
| **Cotrimoxazole resistance - n (%)** | 4/11 (36.4) | 1/2(50) | 3/8 (37.5) | 0.75 |
| **Carbapenem resistance – n (%)** | 2/40 (5) | 1/6 (16.7) | 1/27 (3.7) | 0.23 |

*Some *Campylobacter* spp. BSI included in the table were causes by species other than *C. coli*, *C. jejuni* or *C. fetus*, or the *Campylobacter* species was not reported in the included studies.

**p-value compares PID vs non-PID patients

PID: Primary immunodeﬁciency

**References of case series and case reports included in the systematic review**

1. Shigematsu T, Shibue Y. Bacterial Pericarditis Caused by *Campylobacter fetus* subsp. fetus After Mutton Consumption. Cureus. 2023;15(1):e33213.
2. Katsuno S, Itamoto C, Hase I. Pericarditis due to *Campylobacter coli* infection: a case report. BMC Infect Dis. 2023;23(1):316.
3. Bonilla-Moreno M, Torrecillas M, Laporte-Amargos J, González-Díaz A, Mussetti A, Tubau F, et al. Development of Meropenem Resistance in a Multidrug-Resistant *Campylobacter coli* Strain Causing Recurrent Bacteremia in a Hematological Malignancy Patient. Antimicrob Agents Chemother. 2023;67(7):e0027223.
4. Bastos L, Gomes R, Pocinho S, Baptista T, Mansinho K. *Campylobacter fetus* Cellulitis. Cureus. 2023;15(2):e35328.
5. Adrianza A, Pourfarrokh N, Choi H, Hwang M, Lukey J, Jinadatha C, et al. *Campylobacter coli* bacteremia associated with diarrhea. IDCases. 2023;31:e01734.
6. Su PY, Tan BF, Fu CM, Chen CN, Chou AK, Kung PJ, et al. Concurrence of imatinib-induced massive pleural/pericardial eﬀusion and *Campylobacter* bacteremia in an adolescent with chronic myeloid leukemia. J Infect Chemother. 2022;28(1):103-7.
7. Sorin B, Vigneron J, Fadlallah J, Mondesir J, Fieschi C, Oksenhendler E, et al. *Campylobacter* infection in 4 patients treated with ibrutinib. Eur J Clin Microbiol Infect Dis. 2022;41(5):849-52.
8. Santos-Alonso C, González-García E, Ruíz-Carrascoso G, Loeches-Yagüe B. Bacteremia due to *C. jejuni* in kidney transplant patients. Is immediate post-transplant immunosuppression a risk factor? Enferm Infecc Microbiol Clin. 2022;40(5):275-6.
9. Merrick B, Tamilarasan AG, Luber R, Yong PFK, Cheent K, Irving PM, et al. Recurrent *Campylobacter jejuni* Infection in an Immunodeficient Patient Treated with Repeated Faecal Microbiota Transplant (FMT)—A Case Report. Infect Dis Rep. 2022;14(1):56-62.
10. Jiang L, Gao J, Wang P, Liu Y. Relapsing cellulitis associated with *Campylobacter coli* bacteremia in a Good’s syndrome patient: a case report. BMC Infect Dis. 2022;22(1):354.
11. Itoh N, Akazawa N, Ishibana Y, Hamada S, Hagiwara S, Murakami H. Femoral osteomyelitis caused by oral anaerobic bacteria with mixed bacteremia of *Campylobacter rectus* and Parvimonas micra in a chronic periodontitis patient: a case report. BMC Infect Dis. 2022;22(1):613.
12. Fujita M, Ueno T, Horiuchi M, Mitsuhashi T, Yamamoto S, Arai A, et al. *Campylobacter coli* infection causes spinal epidural abscess with Guillain–Barré syndrome: a case report. BMC Neurol. 2022;22(1).
13. Dobrović K, Fila B, Janeš A, Civljak R. *Campylobacter fetus* Bacteremia Related to Vascular Prosthesis and Pseudoaneurysm Infection: A Case Report and Review. Pathogens. 2022;11(12).
14. Coustillères F, Hanoy M, Lemée L, Le Roy F, Bertrand D. *Campylobacter fetus* bacteremia complicated by multiple splenic abscesses and multivisceral signs in a renal transplant recipient: a case report and review of the literature. Braz J Infect Dis. 2022;26(2):102336.
15. Cameira J, Araújo P, Afonso A, Oliveira D, Ceia F. Renal Abscess and Recurrent Bacteremia Caused by *Campylobacter* Infection in an Adult With Common Variable Immunodeficiency. Cureus. 2022;14(2):e21827.
16. Asatori D, Shimada K. There’s not always one enemy: Co-infection of *Campylobacter* *jejuni* and non- typhoidal salmonella in a patient with systemic lupus erythematosus. Clin Case Rep. 2022;10(11).
17. Adhikari P, Antala D, Bhandari B, Mohamed K, Egoryan G, Stake JJ, et al. A Case of *Campylobacter fetus* Subspecies Fetus Systemic Infection. Cureus. 2022;14(4):e23963.
18. Shionoya K, Tonozuka R, Itoi T, Sofuni A, Tsuchiya T, Ishii K, et al. Severe Acute Cholangitis and Bacteremia Due to *Campylobacter jejuni*: A Case Report and Review of the Literature. Intern Med. 2021;60(23):3737-41.
19. Seong YJ, Lee SH, Kim EJ, Choi YH, Kim TJ, Lee WG, et al. *Campylobacter fetus* subspecies venerealis meningitis associated with a companion dog in a young adult: a case report. BMC Infect Dis. 2021;21(1):1280.
20. Nakatani R, Shimizu K, Matsuo T, Koyamada R, Mori N, Yamashita T, et al. *Campylobacter fetus* bacteremia and meningitis in an acute lymphoblastic leukemia patient undergoing maintenance therapy: a case report. BMC Infect Dis. 2021;21(1).
21. Moﬀatt CRM, Kennedy KJ, Selvey L, Kirk MD. *Campylobacter*-associated hospitalisations in an Australian provincial setting. BMC Infect Dis. 2021;21(1):10.
22. Moﬀatt CRM, Kennedy KJ, O’Neill B, Selvey L, Kirk MD. Bacteraemia, antimicrobial susceptibility and treatment among *Campylobacter*-associated hospitalisations in the Australian Capital Territory: a review. BMC Infect Dis. 2021;21(1).
23. Miyamatsu Y, Tanizaki R, Yamada S, Tsujimura I, Wakabayashi H. Cellulitis with persistent bacteremia caused by *Campylobacter lari* in a patient with mantle-cell lymphoma. IDCases . 2021;23.
24. Kyotani M, Kenzaka T, Akita H, Arakawa S. *Campylobacter insulaenigrae* bacteremia with meningitis: a case report. BMC Infect Dis. 2021;21(1):633.
25. Durovic A, Seth-Smith HMB, Hinic V, Kurz M, Khanna N, Egli A. Two simultaneous cases of disseminated infections with *Campylobacter fetus*: clinical characteristics and molecular comparison. Clin Microbiol Infect. 2021;27(1):141-3.
26. Cole J, Smith S, Huycke M, Fitzgerald R. Case report of *Campylobacter* bacteremia with thrombotic seeding. Crit Care Med. 2021;49(1):333.
27. Awada B, Hindy JR, Chalfoun M, Kanj SS. Cervical osteomyelitis potentially caused by *Campylobacter fetus*. J Infect Public Health. 2021;14(9):1233-6.
28. Roberts SC, Stone SM, Sutton SH, Flaherty JP. Cutaneous Manifestations of *Campylobacter jejun*i Infection: A Case Report and Review of the Literature. Infect Dis Clin Pract. 2020;28(2):61-3.
29. Pisipati S, Zafar A, Zafar Y. *Campylobacter* coli bacteraemia: how common is it? BMJ Case Rep. 2020;13(12).
30. Martora F, Pagliuca C, Della Pepa ME, Della Rocca MT, Curto S, Iovene MR, et al. *Campylobacter jejuni* bacteremia in Italian pediatric patients with acute lymphoblastic leukemia: Report of two cases. New Microbiol. 2020;43(2):96-8.
31. Mariette F, Amrane S, Couteau C, Lagier JC, Eldin C. *Campylobacter jejuni* infection associated with miscarriage, a case report and literature review. J Reprod Immunol. 2020;141.
32. Hartman J, Westerman M, Wagenaar JFP. Two-sided femoral *Campylobacter jejuni* osteomyelitis in a patient with acquired hypogammaglobulinemia: A case report. BMC Infect Dis. 2020;20(1).
33. Gharamti AA, Moukalled N, Taher A, Kanafani ZA. Recurrent *Campylobacter* Bacteremia as the First Manifestation of Hypogammaglobulinemia: a Case Report and Literature Review. Infect Chemother. 2020;52(3):415-20.
34. Ali S, Siddiqui B, Lawal F. *Campylobacter jejuni* prosthetic joint infection in an ulcerative colitis patient in the absence of gastrointestinal symptoms. IDCases. 2020;22.
35. Wu PW, Wang CC. Concurrent *Campylobacter jejuni* bacteremia and intussusception in an immunocompetent five-year-old child. J Microbiol Immunol Infect. 2019;52(2):367-9.
36. Tanabe S, Kutsuna S, Tsuboi M, Takeshita N, Hayakawa K, Ohmagari N. Meningoencephalitis caused by a *Campylobacter fetus* in a patient with chronic alcoholism. Intern Med. 2019;58(15):2247-50.
37. Milito C, Quinti I. Genetic stability of *Campylobacter coli* in patients with primary antibody deficiencies. J Allergy Clin Immunol Pract. 2019;7(5):1707.
38. Isabel R. *Campylobacter jejuni* bacteremia in a patient with asplenia and enteritis. IDCases. 2019;17.
39. Ikeda K, Manabe Y, Fujiwara S, Omote Y, Narai H, Abe K. *Campylobacter fetus* Meningitis and Pyogenic Spondylodiscitis in a Healthy Young Woman. Case Rep Neurol. 2019;11(3):299-303.
40. Genderini FG, Martiny D, Ponthieux F, Argudín MA, Gomez Galdon M, Zaarour A, et al. First case of *Campylobacter rectus* and Solobacterium moorei mixed bacteraemia successfully identified by MALDI TOF-MS. New Microbes New Infect .2019;31.
41. Dion J, Malphettes M, Bénéjat L, Mégraud F, Wargnier A, Boutboul D, et al. *Campylobacter* infection in adult patients with primary antibody deficiency. J Allergy Clin Immunol Pract. 2019;7(3):1038- 1041.e4.
42. Bravo F, Céspedes A, Morales P, Chanqueo L. *Campylobacter jejuni* bacteremia in a patient with HIV infection in AIDS stage. Rev Chilena Infectol. 2019;36(5):663-6.
43. Petridou C, Strakova L, Simpson R. *Campylobacter fetus* prosthetic valve endocarditis presenting as a stroke. JMM Case Rep. 2018;5(5).
44. Oka K, Nakano Y, Sazumi Y, Michitani T, Horiguchi S, Ocho K, et al. Clival osteomyelitis with cavernous sinus thrombosis due to Fusobacterium nucleatum and *Campylobacter rectus* induced by tooth extraction. Intern Med. 2018;57(22):3325-8.
45. Nakazawa H, Nishina S, Sakai H, Ito T, Ishida F, Kitano K. Successful Empiric Therapy for Postsplenectomy Sepsis with *Campylobacter fetus* in an Abattoir Worker with Follicular Lymphoma. Intern Med. 2018;57(22):3329-32.
46. Jawad II, Chandna A, Morris-Jones S, Logan S. Unusual case of Lemierre’s syndrome. BMJ Case Rep. 2018;11(1).
47. Hagiya H, Kimura K, Nishi I, Yoshida H, Yamamoto N, Akeda Y, et al. Emergence of carbapenem non- susceptible *Campylobacter coli* after long-term treatment against recurrent bacteremia in a patient with X-linked agammaglobulinemia. Intern Med. 2018;57(14):2077-80.
48. Cila A, Smith S, Armstrong MR, Hendry SJ, Hanson J. Successful outpatient management of *Campylobacter fetus* bacteremia in an immunocompetent patient with endovascular involvement. IDCases. 2018;14:e00463.
49. Cambier A, Martiny D, Hallin M, Wautier M, Giot JB, Khaldi MZ, et al. *Campylobacter coli* meningitis in a 57-year-old patient. Acta Clin Belg. 2018;73(6):427-30.
50. Yoon JG, Lee SN, Hyun HJ, Choi MJ, Jeon JH, Jung E, et al. *Campylobacter jejuni* Bacteremia in a Liver Cirrhosis Patient and Review of Literature: A Case Study. Infect Chemother. 2017;49(3):230-5.
51. Yamamoto K, Hayakawa K, Nagashima M, Shimada K, Kutsuna S, Takeshita N, et al. Comparison of the clinical and microbiological characteristics of *Campylobacter* and *Helicobacter* bacteremia: the importance of time to blood culture positivity using the BACTEC blood culture systems. BMC Res Notes. 2017;10(1):634.
52. Pierna Álvarez M, de la Fuente del Río R, Gaona Álvarez C, González Velasco C. *Campylobacter coli* bacteremia linked immunosuppression. Rev Lab Clin. 2017;10(2):109-12.
53. Mearelli F, Casarsa C, Breglia A, Biolo G. Septic Shock with Multi Organ Failure Due to Fluoroquinolones Resistant *Campylobacter jejuni*. Am J Case Rep. 2017;18:972-4.
54. Marchand-Senécal X, Bekal S, Pilon PA, Sylvestre JL, Gaudreau C. *Campylobacter fetus* Cluster among men Who Have Sex with Men, Montreal, Quebec, Canada, 2014-2016. Clin Infect Dis. 2017;65(10):1751-3.
55. Lukšić B, Karabuva S, Radić M, Karanović J, Lukšić B. *Campylobacter fetus* Pleuropneumonia and Bacteremia in an Adult Immunocompetent Patient. Clin Microbiol News. 2017;39(18):148-9.
56. Kim Y, Shin JA, Han SB, Cho B, Jeong DC, Kang JH. Recurrent *Campylobacter jejuni* bacteremia in a patient with hypogammaglobulinemia: A case report. Medicine (Baltimore). 2017;96(25):e7238.
57. Josa-Montero D, Bustos-Moya G, Perea-Ronco J, Gualtero-Trujillo S. Bacteremia by *Campylobacter fetus* subsp. fetus in Colombia: Case report. Infection. 2017;21(4):267-9.
58. Fernandes M, Silva S, Spínola Santos A, Lopes Da Silva S, Barbosa M. Cellulitis and *Campylobacter jejuni* bacteremia in a patient with x-linked agammaglobulinemia: Case report. Allergy Eur J Allergy Clin Immunol. 2017;72:254.
59. Dumic I, Sengodan M, Franson JJ, Zea D, Ramanan P. Early Onset Prosthetic Joint Infection and Bacteremia due to *Campylobacter fetus* Subspecies fetus. Case Rep Infect Dis. 2017;2017:5892846.
60. Chávez AC, Barrera S, Leon A, Trueba G. *Campylobacter fetus* Bacteremia in a Healthy Patient Returning from a Trip to the Ecuadorian Amazonia. Zoonoses Public Health. 2017;64(5):391-3.
61. Bhattacharyya RP, Flores EJ, Azar MM. Case 30-2017: A 65-year-old woman with altered mental status, bacteremia, and acute liver failure. New Engl J Med. 2017;377(13):1274-82.
62. Van Samkar A, Brouwer MC, Van Der Ende A, Van De Beek D. *Campylobacter fetus* meningitis in adults report of 2 cases and review of the literature. Medicine. 2016;95(8).
63. Skuhala T, Škerk V, Markotić A, Bukovski S, Desnica B. Septic abortion caused by *Campylobacter jejuni* bacteraemia. J Chemother. 2016;28(4):335-6.
64. Reid MJA, Shannon EM, Baxi SM, Chin-Hong P. Steak tartare endocarditis. BMJ Case Rep. 2016;2016.
65. Li P, Wang M, Li X, Hu F, Song H, Xie Y, et al. Spontaneous bacterial peritonitis caused by oxidase negative *Campylobacter fetus subsp. Testudinum* isolated from the patient with decompensated liver cirrhosis: A case report and literatures review. Int J Clin Exp Med. 2016;9(7):14866-9.
66. Harvala H, Ydring E, Brytting M, Söderblom T, Mäkitalo B, Wallensten A, et al. Increased number of *Campylobacter* bacteraemia cases in Sweden, 2014. Clin Microbiol Infect. 2016;22(4):391-3.
67. Gallo MT, di Domenico EG, Toma L, Marchesi F, Pelagalli L, Manghisi N, et al*. Campylobacter jejuni* fatal sepsis in a patient with non-Hodgkin’s lymphoma: Case report and literature review of a difficult diagnosis. Int J Mol Sci. 2016;17(4).
68. Choi HS, Shin SU, Bae EH, Ma SK, Kim SW. Infectious Spondylitis in a Patient with Chronic Kidney Disease: Identification of *Campylobacter fetus* Subsp. testudinum by 16S Ribosomal RNA Sequencing. Jpn J Infect Dis. 2016;69(6):517-9.
69. Anvarinejad M, Amin Shahidi M, Pouladfar GR, Dehyadegari MA, Mardaneh J. *Campylobacter jejuni*  bacteremia in a Patient With Acute Lymphocytic Leukemia. Iran Red Crescent Med J. 2016;18(6):e23992.
70. Agrawal A, Sikachi RR. Infective abdominal aortitis due to *Campylobacter fetus* bacteremia: A case report and review of literature. Intractable Rare Dis Res. 2016;5(4):290-3.
71. Shinha T. Fatal bacteremia caused by *Campylobacter gracilis*, United States. Emerg Infect Dis. 2015;21(6):1084-5.
72. Nakamura I, Omori N, Umeda A, Ohkusu K, Matsumoto T. First case report of fatal sepsis due to *Campylobacter upsaliensis*. J Clin Microbiol. 2015;53(2):713-5.
73. Melendez BA, Hollis HWJ, Rehring TF. Mycotic popliteal aneurysm rupture secondary to *Campylobacter fetus*. Ann Vasc Surg. 2015;29(1):122.e9-11.
74. Kweon OJ, Lim YK, Yoo B, Kim HR, Kim TH, Lee MK. First Case Report of *Campylobacter volucris* Bacteremia in an Immunocompromised Patient. J Clin Microbiol. 2015;53(6):1976-8.
75. Kuperman-Shani A, Vaknin Z, Mendlovic S, Zaidenstein R, Melcer Y, Maymon R. *Campylobacter coli* infection causing second trimester intrauterine growth restriction (IUGR): a case report and review of the literature. Prenat Diagn. 2015;35(12):1258-61.
76. Iraola G, Betancor L, Calleros L, Gadea P, Algorta G, Galeano S, et al. A rural worker infected with a bovine-prevalent genotype of *Campylobacter fetus subsp. fetus* supports zoonotic transmission and inconsistency of MLST and whole-genome typing. Eur J Clin Microbiol Infect Dis. 2015;34(8):1593- 6.
77. Hagiya H, Ogawa H, Takahashi Y, Hasegawa K, Hanayama Y, Otsuka F. Infective internal iliac artery aneurysm caused by *Campylobacter fetus*. Intern Med. 2015;54(16):2021-4.
78. Ghimire R, Urban C, Lee A, Pokhrel A, Wehbeh W, Turett G. *Campylobacter fetus* infection of the aorta: A case report and review of literature. Am J Infect Dis. 2015;11(2):26-32.
79. Duarte Ferreira R, Silva SP, Branco Ferreira M, Lopes Silva S, Barbosa MP. Para-infectious pericarditis as rare complication in X-linked agammaglobulinemia. Allergy Eur J Allergy Clin Immunol. 2015;70:371-2.
80. Yamagami K, Miyashita T, Nakamura T, Shirano M, Nakamura T, Kameda K, et al. *Campylobacter fetus* bacteremia with purulent pleurisy in a young adult with primary hypogammaglobulinemia. Intern Med. 2014;53(11):1221-5.
81. Teglia O, Borda N, García A, Notario R. Recurrent *Campylobacter fetus* bacteriemia in a patient without immunosuppression. Salud Ci. 2014;20(7):761-2.
82. Ruiz-Castillo A, González-Estrada A, Giráldez-Gallego A, Lepe-Jiménez JA. Spontaneous bacteremia due to *Campylobacter coli* in a cirrhotic patient. Enferm Infecc Microbiol Clin Monogr. 2014;32(7):470-1
83. Rodríguez-Alvarez R, Goikoetxea J, García M, Figueras Y, Hernández L, Montejo M. Endovascular *Campylobacter* infections: report of two cases. Rev Esp Quimioter. 2014;27(2):127-9.
84. Pereira L, Sampaio S, Tavares I, Bustorﬀ M, Pestana M. Bacteremia due to *Campylobacter* in renal transplantation: a case report and review of literature. Transpl Infect Dis. 2014;16(6):1007-1
85. Mikals K, Masel J, Gleeson T. *Campylobacter fetus* bacteremia in an immunocompetent traveler. Am J Trop Med Hyg. 2014;91(4):766.
86. Leo QJN, Bolger DTJ. Septic cavernous sinus thrombosis due to *Campylobacter rectus* infection. BMJ Case Rep. 2014;2014.
87. Alnimr AM. A case of bacteremia caused by *Campylobacter fetus*: An unusual presentation in an infant. Infect Drug Resist. 2014;7:37-40.
88. Suy F, Dû DL, Roux AL, Hanachi M, Dinh A, Crémieux AC. Meningitis and endocarditis caused by *Campylobacter fetus* after raw-liver ingestion. J Clin Microbiol. 2013;51(9):3147-50
89. Shumyak L, Damisse PR, Sandin RL, Greene JN. Thirty-eight-year-old woman presenting with fever, diarrhea, and bacteremia. Infect Dis Clin Pract. 2013;21(5):312-3.
90. Prendki V, Marmor S, Zeller V, Lhotellier L, Mégraud F, Desplaces N. *Campylobacter infection* after prosthetic joint surgery. Scand J Infect Dis. 2013;45(9):706-10.
91. Nagy MT, Hla SM. *Campylobacter fetus* sepsis in an immunocompetent patient with haematological complication. BMJ Case Rep. 2013.
92. Martinez-Balzano C, Kohlitz PJ, Chaudhary P, Hegazy H. *Campylobacter fetus* bacteremia in a young healthy adult transmitted by khat chewing. J Infect. 2013;66(2):184-6.
93. Hadano Y, Iwata H. An unusual cause of spontaneous bacterial peritonitis due to *Campylobacter fetus* with alcoholic liver cirrhosis. BMJ Case Rep . 2013.
94. Ariganello P, Angelino G, Scarselli A, Salfa I, Della Corte M, De Matteis A, et al. Relapsing *Campylobacter jejuni* Systemic Infections in a Child with X-Linked Agammaglobulinemia. Case Rep Pediatr. 2013;2013:735108.
95. Shimizu Y, Ishii A, Takahata A, Kajiyama T, Yamahatsu A, Io H, et al. *Campylobacter* bacteremia in hemodialysis patients by eating raw meat - the importance of sanitary education. Case Rep Nephrol Urol. 2012;2(2):145-51.
96. Sakran W, Levin C, Kenes Y, Colodner R, Koren A. Clinical spectrum of serious bacterial infections among splenectomized patients with hemoglobinopathies in Israel: A 37-year follow-up study. Infection. 2012;40(1):35-9.
97. Louwen R, van Baarlen P, van Vliet AHM, van Belkum A, Hays JP, Endtz HP. *Campylobacter* bacteremia: a rare and under-reported event? Eur J Microbiol Immunol (Bp). 2012;2(1):76-87.
98. Liu P. Campylobacteremia in stage IV gliosarcoma with bevacizumab treatment. J Community Hosp Intern Med Perspect. 2012;2(1).
99. Ledina D, Ivić I, Karanović J, Karanović N, Kuzmičić N, Ledina D, et al. *Campylobacter fetus* infection presenting with bacteremia and cellulitis in a 72-year-old man with an implanted pacemaker: A case report. J Med Case Rep. 2012;6.
100. Bessede E, Labadie L, Bakiri S, Lehours P, Megraud F. Comparison of risk factors for *Campylobacter jejuni*, *Campylobacter coli* and *Campylobacter foetus* isolated in France. Clin Microbiol Infect. 2012;18:124.
101. Abe I, Nomura M, Watanabe M, Shimada S, Kohno M, Matsuda Y, et al. Pheochromocytoma crisis caused by *Campylobacter fetus*. Int J Urol. 2012;19(5):465-7.
102. Meyer A, Theulin A, Chatelus E, Sordet C, Javier RM, Chiﬄot H, et al. *Campylobacter fetus* infection in three patients treated with rituximab for rheumatoid arthritis. Arthritis Care Res. 2011;63(10).
103. Lam JYW, Wu AKL, Ngai DC, Teng JLL, Wong ESY, Lau SKP, et al. Three cases of severe invasive infections caused by *Campylobacter rectus* and first report of fatal *C. rectus* infection. J Clin Microbiol. 2011;49(4):1687-91.
104. Haruyama A, Toyoda S, Kikuchi M, Arikawa T, Inami S, Otani N, et al. *Campylobacter fetus* as cause of prosthetic valve endocarditis. Tex Heart Inst J. 2011;38(5):584-7.
105. Brah S, Chiche L, Brun M, Schleinitz N, Harle JR, Durand JM. *Campylobacter fetus* Bacteremia Revealed by Cellulitis without Gastrointestinal Symptoms in the Context of Acquired Hypogammaglobulinemia: A Report of Three Cases. Case Rep Gastrointest Med. 2011;2011:628902.
106. Aguadero V, García AM, Sánchez J, Sánchez JL. Fatal bacteremia caused by *Campylobacter fetus* and *Campylobacter jejuni* in patients with alcoholic liver disease. Rev Esp Quimioter. 2011;24(3):166-7.
107. Van den Bruele T, Mourad-Baars PEC, Claas ECJ, van der Plas RN, Kuijper EJ, Bredius RGM. *Campylobacter jejuni* bacteremia and *Helicobacter pylori* in a patient with X-linked agammaglobulinemia. Eur J Clin Microbiol Infect Dis. 2010;29(11):1315-9.
108. Romero Gómez MP, García-Perea A, Ruiz Carrascoso G, Bajo MA, Mingorance J. *Campylobacter fetus* peritonitis and bacteremia in a patient undergoing continuous ambulatory peritoneal dialysis. J Clin Microbiol. 2010;48(1):336-7.
109. Lemaire X, Dehecq C, Cattoen C, Garnier LD, Bournet BS, Yazdanpanah Y, et al. Spondylodiscitis and an aortic aneurysm due to *Campylobacter coli*. Ann Clin Microbiol Antimicrob. 2010;9:8.
110. Aggarwal H, Kushnir L, Conti D, Gallichio M, Tobin E. A case of *Campylobacter jejuni* bacteremia in a renal transplant patient. Transpl Infect Dis. 2010;12(6):518-20.
111. Wong JSJ, Anderson TP, Chambers ST, On SLW, Murdoch DR. *Campylobacter fetus*-associated epidural abscess and bacteremia. J Clin Microbiol. 2009;47(3):857-8.
112. Shiferson A, Ascher E, Hingorani A, Puggioni A, Marks N, Tran V, et al. Bilateral internal iliac artery aneurysm infected with *Campylobacter fetus*. Vascular. 2009;17(4):226-9.
113. Roan JN, Ko WC, Luo CY. Abdominal septic aortic pseudoaneurysm caused by *Campylobacter jejuni* infection: report of a case. Surg Today. 2009;39(2):137-40.
114. Okada H, Kitazawa T, Harada S, Itoyama S, Hatakeyama S, Ota Y, et al. Combined treatment with oral kanamycin and parenteral antibiotics for a case of persistent bacteremia and intestinal carriage with *Campylobacter coli.* Intern Med. 2008;47(14):1363-6.
115. Ahmar W, Johnson D, Richards M, Strathmore N. *Campylobacter fetus* infection of an internal cardioverter defibrillator. PACE Pacing Clin Electrophysiol. 2008;31(2):258-9.
116. Malani AN, Malani PN, Cinti SK. Campylobacter fetus: An unusual cause of prosthetic joint infection. Infect Dis Clin Pract. 2007;15(2):119-21.
117. Chua K, Gürtler V, Montgomery J, Fraenkel M, Mayall BC, Grayson ML. *Campylobacter insulaenigrae* causing septicaemia and enteritis. J Med Microbiol. 2007;56(11):1565-7.
118. Arai A, Kitano A, Sawabe E, Kanegane H, Miyawaki T, Miura O. Relapsing *Campylobacter coli* bacteremia with reactive arthritis in a patient with X-linked agammaglobulinemia. Intern Med. 2007;46(9):605-9.
119. Rajendran PM, Hurst E, Ruben B, Maurer T. *Campylobacter* presenting as erythematous plaques on the lower extremity of a man with human immunodeficiency virus [10]. Arch Dermatol. 2006;142(9):1240-1.
120. Kopecký O, Lukešová Š, Horáček J, Pařízková R. *Campylobacter* sepsis with multiple organ failure in IgG subclass deficiency. Folia Microbiol. 2006;51(6):604-8.
121. Jirapongsananuruk O, Wanotayan K, Phongsamart W, Chokephaibulkit K, Visitsunthorn N, Luangwedchakarn V, et al. Recurrent *Campylobacter lari* bacteremia in X-linked agammaglobulinemia: a case report and review. Asian Pac J Allergy Immunol. 2006;24(2):171-4.
122. Fica C A, Illanes R V, Sakurada Z A, Vidal C M, Valenzuela M ME. Bacteraemia due to *Campylo bacter*  *fetus* in an immune suppressed patient. Rev Chilena Infectol. 2006;23(4):336-9.
123. Borda N, Gambande T, Notario R. Two cases of enteritis with bacteremia due to *Campylobcter jejuni*. Medicina (Argentina). 2006;66(5):450-2.
124. Zonios DI, Panayiotakopoulos GD, Kabletsas EO, Tzima EL, Stefanou I, Archimandritis AJ. *Campylobacter fetus* bacteraemia in a healthy individual: clinical and therapeutical implications. J Infect. 2005;51(4):329-32.
125. Smithson-Amat A, Perelló-Carbonell R, Miret-Mas C, Rodríguez-Flores E, Bastida-Vila MT, Nolla-Salas. M. Spontaneous bacterial empyema due to *Campylobacter jejuni*. Gastroenterol Hepatol. 2005;28(8):509.
126. Ruiz P, Obregón F, Ortiz De Zárate J, Cabezudo P, Polo F, Marcé L, et al. *Campylobacter jejuni* bacteriemia associated with acute pancreatitis [5]. Rev Esp Enferm Dig. 2005;97(5):383-4.
127. Miki K, Maekura R, Hiraga T, Hirotani A, Hashimoto H, Kitada S, et al. Infective tricuspid valve endocarditis with pulmonary emboli caused by *Campylobacter fetus* after tooth extraction. Intern Med. 2005;44(10):1055-9.
128. Tokuda K, Nishi J, Miyanohara H, Sarantuya J, Iwashita M, Kamenosono A, et al. Relapsing cellulitis associated with *Campylobacter coli* bacteremia in an agammaglobulinemic patient. Pediatr Infect Dis J. 2004;23(6):577-9.
129. Teh HS, Chiang SH, Tan AGS, Sng LH, Oh HML. A case of right loin pain: Septic ovarian vein thrombosis due to *Campylobacter fetus* bacteraemia. Ann Acad Med Singapore. 2004;33(3):385-8.
130. Rollot K, Albert JD, Werner S, Tattevin P, Cozic I, Perdriger A, et al. *Campylobacter fetus* septic arthritis revealing a malignancy. Jt Bone Spine. 2004;71(1):63-5.
131. Rodríguez-Vázquez M, García-Arpa M, Porras-Leal L, Romero-Aguilera G, Cortina-De La Calle P, Romero-Aguilera MD. Atypical cellulitis associated with bacteremia from *Campylobacter jejuni* in a patient with X-linked hypogammaglobulinemia. Actas Dermo-Sifiliogr. 2004;95(1):41-3.
132. Monselise A, Blickstein D, Ostfeld I, Segal R, Weinberger M. A case of cellulitis complicating *Campylobacter jejuni* subspecies jejuni bacteremia and review of the literature. Eur J Clin Microbiol Infect Dis. 2004;23(9):718-21.
133. Herve J, Aissa N, Legrand P, Sorkine M, Calmette MJ, Santin A, et al. *Campylobacter fetus* meningitis in a diabetic adult cured by imipenem. Eur J Clin Microbiol Infect Dis. 2004;23(9):722-4.
134. Remacha MaA, Esteban A, González-Castañeda C, Fernández-Natal I, Echeita A. *Campylobacter fetus*  bacteremia in immunocompetent patient . An Med Interna. 2003;20(8):439-40.
135. Chuman Y, Takata T, Sameshima H, Takeuchi S, Takatsuka Y, Makino T, et al. *Campylobacter fetus* bacteremia in a patient with adult T cell leukemia. Clin Infect Dis. 2003;36(11):1497-8.
136. Werno AM, Klena JD, Shaw GM, Murdoch DR. Fatal case of *Campylobacter lari* prosthetic joint infection and bacteremia in an immunocompetent patient. J Clin Microbiol. 2002;40(3):1053-5.
137. Rafi A, Matz J. An unusual case of *Campylobacter jejuni* pericarditis in a patient with X-linked agammaglobulinemia. Ann Allergy Asthma Immunol. 2002;89(4):362-7
138. Oshikiri T, Morikawa T, Sugiura H, Katoh H. Thymoma associated with hypogammaglobulinemia (Good’s syndrome): report of a case. Surg Today. 2002;32(3):264-6.
139. Lau SKP, Woo PCY, Leung KW, Yuen KY. Emergence of cotrimoxazole- and quinolone-resistant *Campylobacter* infections in bone marrow transplant recipients. Eur J Clin Microbiol Infect Dis. 2002;21(2):127-9.
140. Krause R, Ramschak-Schwarzer S, Gorkiewicz G, Schnedl WJ, Feierl G, Wenisch C, et al. Recurrent septicemia due to *Campylobacter fetus* and *Campylobacter lari* in an immunocompetent patient. Infection. 2002;30(3):171-4.
141. Heng AE, De Champs C, Souweine B, Guy L, Sirot J, Deteix P. *Campylobacter fetus* bacteraemia in a renal graft recipient. Nephrol Dial Transplant. 2002;17(4):689-90.
142. Briedis DJ, Khamessan A, McLaughlin RW, Vali H, Panaritou M, Chan ECS. Isolation of *Campylobacter fetus subsp. fetus* from a patient with cellulitis. J Clin Microbiol. 2002;40(12):4792-6.
143. Akiba T, Akiba K, Suto N, Kumagai KI, Sakamoto M, Yazaki N. *Campylobacter coli* bacteremia in an 11-year-old boy. Pediatr Int. 2002;44(5):543-4.
144. Viejo G, Gomez B, De Miguel D, Del Valle A, Otero L, De La Iglesia P. *Campylobacter fetus* *subespecies fetus* bacteremia associated with chorioamnionitis and intact fetal membranes. Scand J Infect Dis. 2001;33(2):126-7
145. Mahe I, Perdrix C, Maniere T, Holeman A, Diemer M, Bergmann JF. A case of *Campylobacter fetus* endocarditis of the tricuspid valve unaccompanied by fever . Am J Med. 2001;111(5):418.
146. Maestre JR, Buezas V, Sánchez P, Montero A, Mellado F. Bacteremia caused by *Campylobacter jejuni* in a 22 year old male with autoimmune heamolytic anaemia. Enferm Infecc Microbiol Clin. 2001;19(9):457-8.
147. Cesteros RI, Menasalvas AI, Guerrero C, López-Domínguez R, Miranda A, Segovia M. Bacteremia due *to Campylobacter jejuni:* report of two cases. Enferm Infecc Microbiol Clin. 2001;19(4):189-90.
148. Anstead GM, Jorgensen JH, Craig FE, Blaser MJ, Patterson TF. Thermophilic multidrug-resistant *Campylobacter fetus* infection with hypersplenism and histiocytic phagocytosis in a patient with acquired immunodeficiency syndrome. Clin Infect Dis. 2001;32(2):295-6.
149. Vandewal W. Report a case of *Campylobacter coli* bacteremia in a patient with gastro-enteritis. Acta Clin Belg. 2000;55(6):344.
150. Peetermans WE, De Man F, Moerman P, van de Werf F. Fatal prosthetic valve endocarditis due to *Campylobacter fetus*. J Infect. 2000;41(2):180-2.
151. Kaneko M, Watanabe J, Ueno E, Nakano N. *Campylobacter jejuni* bacteremia in an immunocompetent Japanese child. Pediatr Int. 2000;42(5):579-81.
152. Bucknell SJ, Le T, Amerena J, Hill DG, McDonald M. Aortic dissection associated with *Campylobacter* aortitis. Heart Lung Circul. 2000;9(2):88-91.
153. Adedeji A, Subudhi CP, Gokal R, Hutchison AJ, Kerr JR. *Campylobacter jejuni* bacteremia, peritonitis, and exacerbation of chronic pancreatitis in a patient on CAPD: case report and literature review. Perit Dial Int. 2000;20(6):794-6.
154. Sakran W, Raz R, Levi Y, Colodner R, Koren A. *Campylobacter* bacteremia and pneumonia in two splenectomized patients. Eur J Clin Microbiol Infect Dis. 1999;18(7):496-8.
155. Manfredi R, Nanetti A, Ferri M, Chiodo F. Fatal *Campylobacter jejuni* bacteraemia in patients with AIDS. J Med Microbiol. 1999;48(6):601-3.
156. Lozano P, Rimbau EM, Martinez S, Ribas AM, Gómez FT. *Campylobacter fetus* infection of a previously excluded popliteal aneurysm. Eur J Vasc Endovasc Surg. 1999;18(1):86-8.
157. Burch KL, Saeed K, Sails AD, Wright PA. Successful treatment by meropenem of *Campylobacter jejuni*  meningitis in a chronic alcoholic following neurosurgery. J Infect. 1999;39(3):241-3.
158. Tee W, Luppino M, Rambaldo S. Bacteremia due to *Campylobacter sputorum* biovar sputorum. Clin Infect Dis. 1998;27(6):1544-5.
159. Morris CN, Scully B, Garvey GJ. Campylobacter lari associated with permanent pacemaker infection and bacteremia. Clin Infect Dis. 1998;27(1):220-1.
160. Meier PA, Dooley DP, Jorgensen JH, Sanders CC, Huang WM, Patterson JE. Development of quinolone-resistant *Campylobacter fetus* bacteremia in human immunodeficiency virus-infected patients. J Infect Dis. 1998;177(4):951-4.
161. Maccario M, Tarantino A, Nobile-Orazio E, Ponticelli C. *Campylobacter jejuni* bacteremia and Guillain-Barré syndrome in a renal transplant recipient. Transpl Int. 1998;11(6):439-42.
162. Ichiyama S, Hirai S, Minami T, Nishiyama Y, Shimizu S, Shimokata K, et al. *Campylobacter fetus subspecies fetus* cellulitis associated with bacteremia in debilitated hosts. Clin Infect Dis. 1998;27(2):252-255+256-258.
163. Dronda F, García-Arata I, Navas E, De Rafael L. Meningitis in adults due to *Campylobacter fetus* subspecies fetus. Clin Infect Dis. 1998;27(4):906-7.
164. Callahan C, Greene JN, Sandin RL, Ruge D, Johnson J. *Campylobacter jejuni* bacteremia in an HIV- positive patient with non- Hodgkin’s lymphoma. Cancer Control. 1998;5(4):357-60.
165. Schuster M, Blaser MJ, Nachamkin I. Serendipitous detection of persistent *Campylobacter jejuni* *subspecies jejuni* bacteremia in a patient undergoing bone marrow transplantation. Clin Infect Dis. 1997;24(6):1270.
166. Ruiz-Contreras J, Ramos JT, Hernández-Sampelayo T, De José M, Clemente J, Gurbindo MD, et al. *Campylobacter* sepsis in human immunodeficiency virus-infected children. Pediatr Infect Dis J. 1997;16(2):251-3.
167. Korman TM, Varley CC, Spelman DW. Acute hepatitis associated with *Campylobacter jejuni* bacteraemia. Eur J Clin Microbiol Infect Dis. 1997;16(9):678-81.
168. Jackson N, Zaki M, Rahman AR, Nazim M, Win MN, Osman S. Fatal *Campylobacter jejuni* infection in a patient splenectomised for thalassaemia. J Clin Pathol. 1997;50(5):436-7.
169. Hsueh PR, Teng LJ, Yang PC, Ho SW, Luh KT. Indwelling device-related bacteremia caused by serum- susceptible *Campylobacter coli*. J Clin Microbiol. 1997;35(8):2178-80.
170. Meyrieux V, Monneret G, Lepape A, Chomarat M, Banssillon V. Fatal septic shock with multiple organ failure due to *Campylobacter jejuni.* Clin Infect Dis. 1996;22(1):183-4.
171. Watine J, Martorell J, Bruna T, Gineston JL, Poirier JL, Lamblin G. In vivo peﬂoxacin-resistant *Campylobacter fetus* responsible for gastro-intestinal infection and bacteremia associated with arthritis of the hip. Yonsei Med J. 1995;36(2):202-5.
172. Molina JM, Casin I, Hausfater P, Giretti E, Welker Y, Decazes JM, et al. *Campylobacter* infections in HIV-infected patients: Clinical and bacteriological features. AIDS. 1995;9(8):881-5.
173. Brmbolic B. Multiple abscesses of the liver caused by *Campylobacter jejuni*. J Clin Gastroenterol.1995;20(4):307-9.
174. Armstrong C, Murphy PG. Bacteraemia caused by *Campylobacter* spp. J Clin Patho. 1995;48(6):596. 175.
175. Yamazaki K, Watanabe N, Hasegawa A, Yamaguchi E, Miyamoto K, Sakiyama Y, et al. Good’s syndrome with a block in the early stage of B cell diﬀerentiation and complicated by *Campylobacter fetus* sepsis. Intern Med. 1994;33(8):496-500.
176. Neuzil KM, Wang E, Haas DW, Blaser MJ. Persistence of *Campylobacter fetus* bacteremia associated with absence of opsonizing antibodies. J Clin Microbiol. 1994;32(7):1718-20.
177. Ladron de Guevara C, Gonzalez J, Pena P. Bacteraemia caused by *Campylobacter spp.* J Clin Pathol.1994;47(2):174-5.
178. Harvey P, Bayardelle P, Bélanger R, Fortin L. Sacroiliitis and septicemia caused by *Campylobacter rectus* and *Actinomyces odontolyticus*. Can J Infect Dis. 1994;5(3):133-6.
179. Sauerwein RW, Bisseling J, Horrevorts AM. Septic abortion associated with *Campylobacter fetus subspecies fetus* infection: Case report and review of the literature. Infection. 1993;21(5):331-3.
180. Peterson MC, Farr RW, Castiglia M. Prosthetic hip infection and bacteremia due to *Campylobacter jejuni* in a patient with AIDS. Clin Infect Dis. 1993;16(3):439-40.
181. Nelson MR, Shanson DC, Hawkins DA, Gazzard BG. *Salmonella*, *Campylobacter* and *Shigella* in HIV- seropositive patients. AIDS. 1992;6(12):1495-8.
182. Kerstens PJSM, Endtz HP, Meis JFGM, Oyen WJG, Koopman RJI, Van Den Broek PJ, et al. Erysipelas- like skin lesions associated with *Campylobacter jejuni* septicemia in patients with hypogammaglobulinemia. Eur J Clin Microbiol Infect Dis. 1992;11(9):842-7
183. Ezpeleta C, de Ursua PR, Obregon F, Goñi F, Cisterna R. Acute pancreatitis associated with *Campylobacter jejuni* bacteremia. Clin Infect Dis. 1992;15(6):1050.
184. Allerberger F, Kasten MJ, Anhalt JP. *Campylobacter* fetus subspecies fetus infection. Klin Wochenschr. 1991;69(17):813-6
185. Morrison VA, Lloyd BK, Chia JK, Tuazon CU. Cardiovascular and bacteremic manifestations of *Campylobacter fetus* infection: case report and review. Rev Infect Dis. 1990;12(3):387-92.
186. Chusid MJ, Wortmann DW, Dunne WM. *Campylobacter upsaliensis* sepsis in a boy with acquired hypogammaglobulinemia. Diagn Microbiol Infect Dis. 1990;13(5):367-9.
187. Zhong Y,  [Wu](https://pubmed.ncbi.nlm.nih.gov/?term=Wu+J&cauthor_id=39208964) J, [Liu](https://pubmed.ncbi.nlm.nih.gov/?term=Liu+L&cauthor_id=39208964) L , [Luo](https://pubmed.ncbi.nlm.nih.gov/?term=Luo+J&cauthor_id=39208964)  J, [Xiong](https://pubmed.ncbi.nlm.nih.gov/?term=Xiong+X&cauthor_id=39208964)  X, [Wang](https://pubmed.ncbi.nlm.nih.gov/?term=Wang+G&cauthor_id=39208964)  G, [Zhou](https://pubmed.ncbi.nlm.nih.gov/?term=Zhou+Y&cauthor_id=39208964) Y. Characterization of a ST137 multidrug-resistant *Campylobacter jejuni* strain with a tet(O)-positive genomic island from a bloodstream infection patient. Microb Pathog 2024 Oct:195:106900.  doi: 10.1016/j.micpath.2024.106900. Epub 2024 Aug 28.
188. Ding, C.H, Wahab, A.A., Tzar, M.N., Mokhtar, M.N., Arunasalam, V. *Campylobacter jejuni* bacteremia in a leukemic child: a nearly missed diagnosis. *Tropical Biomedicine* 41(2): 206-208 (2024). Doi: <https://doi.org/10.47665/tb.41.2.011>.
189. García-Sánchez C, García-Rodríguez J, Ruiz-Carrascoso G. Clinical and microbiological findings of recurrent *Campylobacter* spp. gastroenteritis in a tertiary care hospital. Enfermedades Infecciosas y Microbiología Clínica 42 (2024) 257–262. Doi: 10.1016/j.eimce.2022.09.013.
190. Beery J, Roberston K, Hynes A, Douglas A, Peters J, Freedle R, Chamberland R, Reilly K, Abate G. *Campylobacter* gastroenteritis and bacteremia in an asplenic patient with a recent history of Yersinia Enterocolitis: Case report and literature review. IDCases 36 (2024) e01984. <https://doi.org/10.1016/j.idcr.2024.e01984>.
191. Nakamura I, Kobayashi T, Fukuzawa M, Komori K, Yamaguchi T. Cellulitis with bacteremia due to multidrug-resistant *Campylobacter jejuni* in a case of agammaglobulinemia and bronchiectasis. IDCases 37 (2024) e02010. https://doi.org/10.1016/j.idcr.2024.e02010 .
192. Bak A, Kim TS, Park H,Park JH. Prosthetic valve endocarditis caused by *Campylobacter fetus*: a case report and literature review. Journal of International Medical Research 2023, Vol. 51(11) 1–9. DOI: 10.1177/03000605231213264.
193. Grouteau G, Mignonat C, Marchou B, Martin-Blondel G, Glass O, Roubaud-Baudron C, et al. *Campylobacter fetus* foodborne illness outbreak in the elderly. Front Microbiol [Internet]. 2023;14. Doi: 10.3389/fmicb.2023.1194243.
194. Lenherr A, Boughdad S, Prior JO, Lalonde MN, Filippidis P. Infective aortitis and subacute myocarditis due to *Campylobacter fetus*. Int J Infect Dis 2024 Jan:138:113-114. doi: 10.1016/j.ijid.2023.11.033.
195. Gaultier S, ,Jousset AB, Soudani M, Durroux A, Mihaila L, Neiss M, Collarino R, Jaureguiberry S, Escaut L. *Campylobacter coli* enteritis associated with *Campylobacter fetus* bacteremia, spondylodiscitis, and late CIED-related endocarditis, a case report. Heliyon 10 (2024) e24418. <https://doi.org/10.1016/j.heliyon.2024.e24418>.
196. Uehara H, Oe Y, Yoshimura T, Gunji T , Okuyama M. Acute Cholecystitis Caused by *Campylobacter jejuni* Mimicking Acute Coronary Syndrome. Cureus 2024. 16(2): e53608. DOI 10.7759/cureus.53608.

**Supplemental Figure S1.** Funnel plots for the meta-analysis of mortality associated with *Campylobacter* spp. bloodstream infection.

**
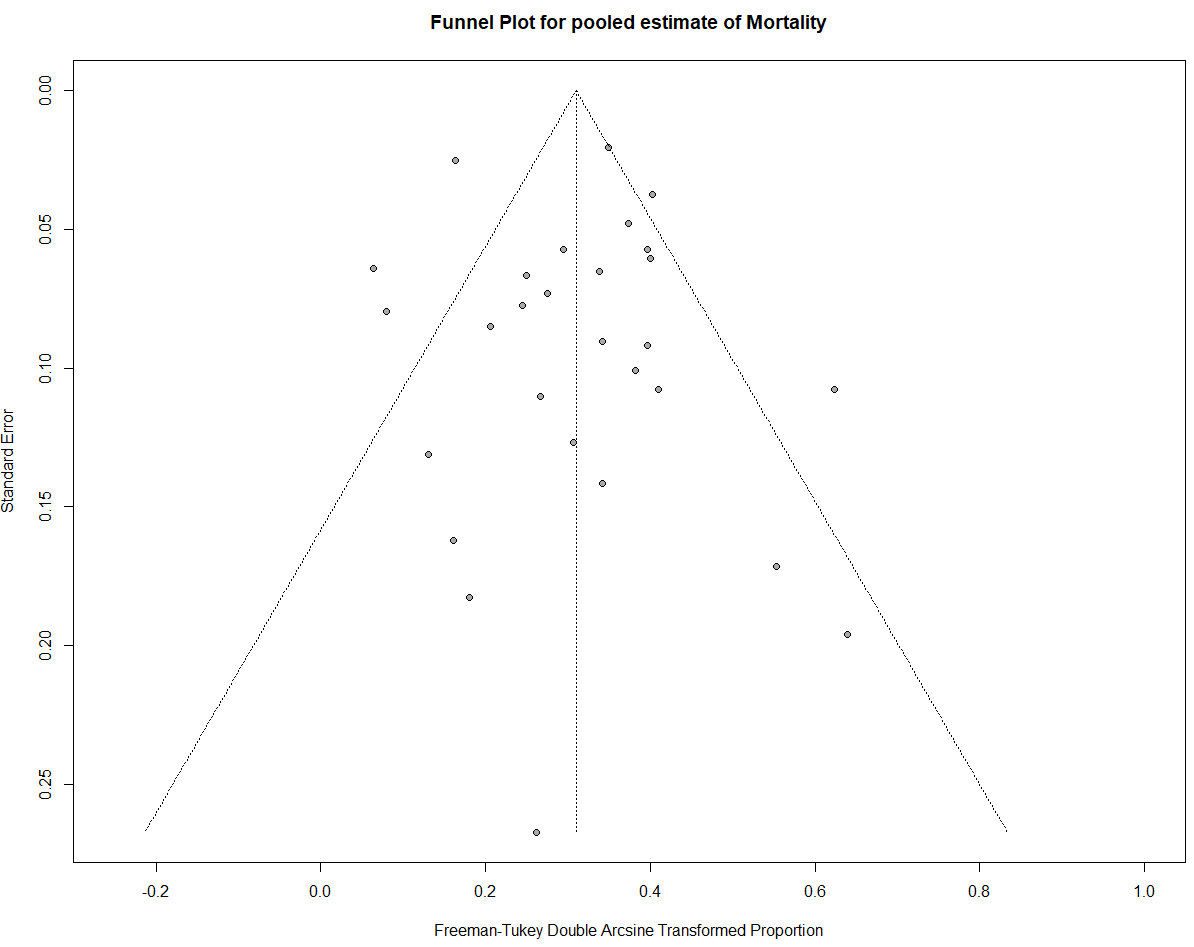
**

**
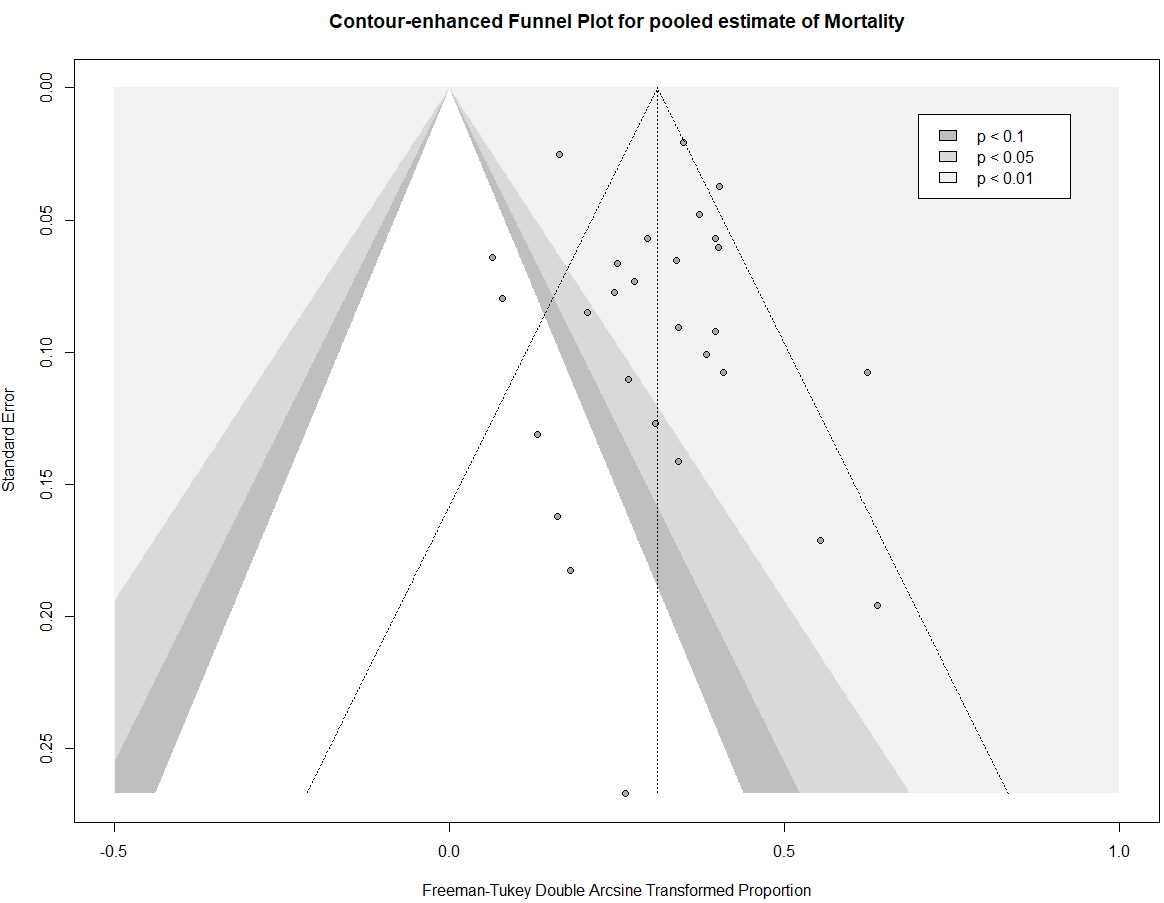
**

**Supplemental Figure S2.** Meta-analysis for the Mortality associated with *Campylobacter* spp. bloodstream infection using Trim-and-fill method to adjust for funnel plot asymmetry.

**
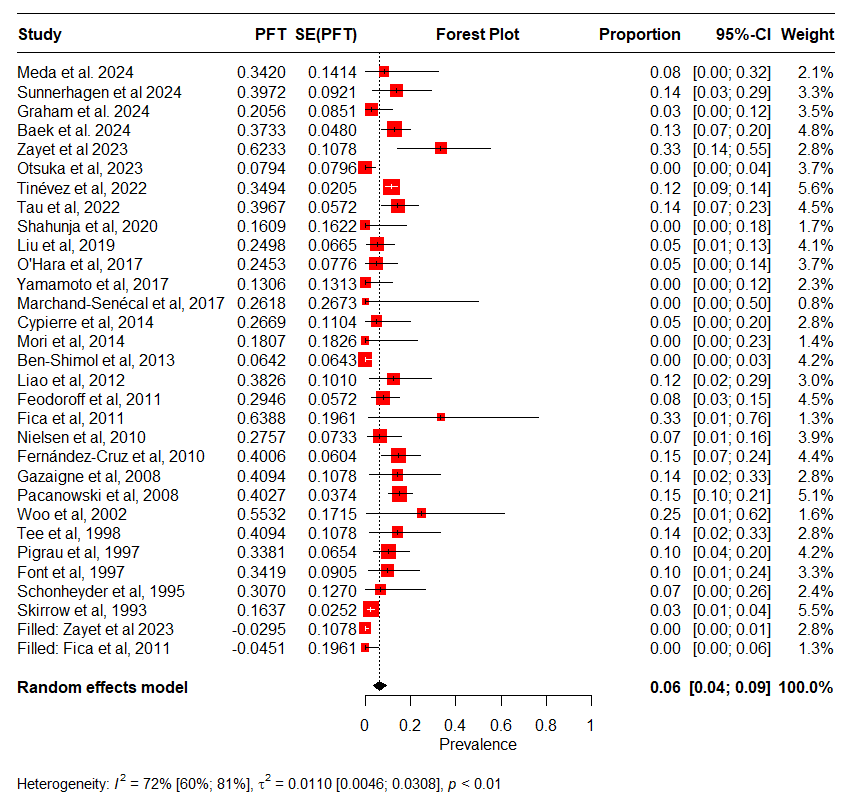
**

**Supplemental Figure S3.** Funnel plots for the meta-analysis of relapse associated with *Campylobacter* spp. bloodstream infection.

**
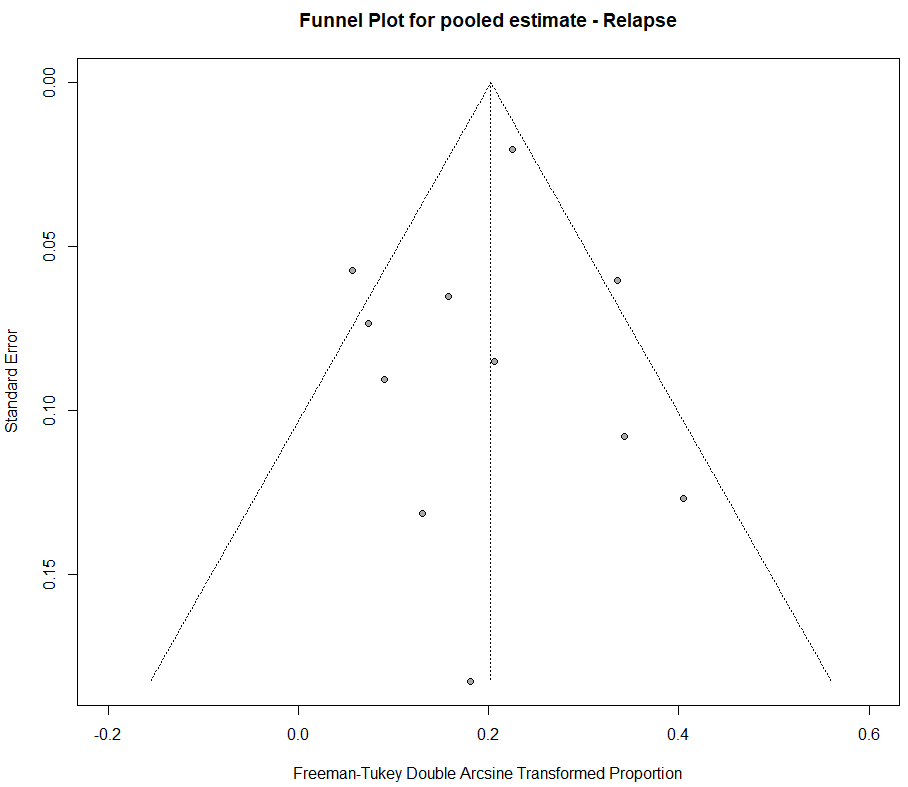
**

**
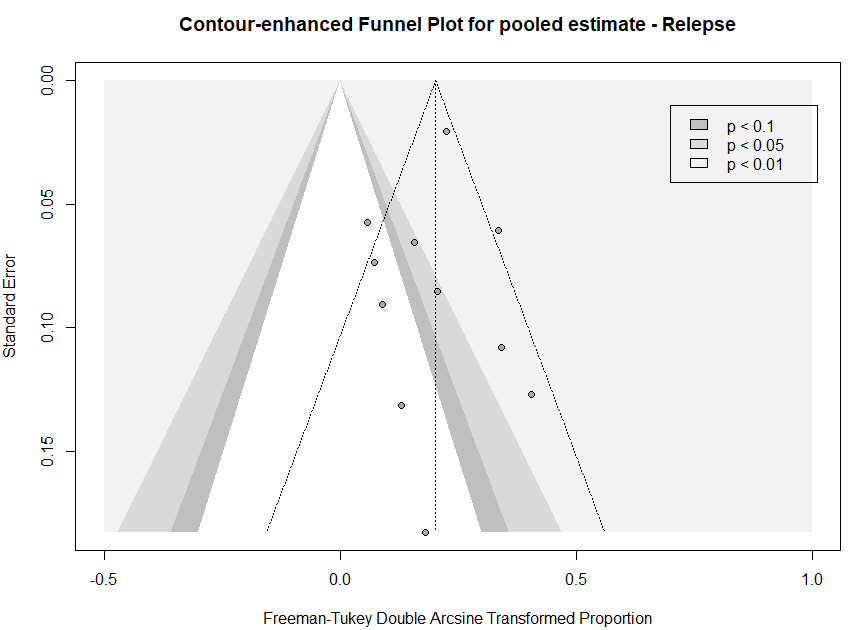
**
